# ‘Us and them’: A realist interview study exploring how and why health system factors influence dentists’ participation in state funded, contracted primary dental care for low-income populations in Ireland

**DOI:** 10.1371/journal.pone.0341786

**Published:** 2026-07-31

**Authors:** Paul Leavy, Blánaid Daly, John Ford, Sara Burke

**Affiliations:** 1 Centre for Health Policy and Management, Trinity College Dublin, Dublin 2, Ireland; 2 School of Dental Science, Trinity College Dublin, Dublin 2, Ireland; 3 Wolfson Institute of Population Health, Queen Mary University of London, London, United Kingdom; University of Insubria, ITALY

## Abstract

**Objectives:**

To identify and explain the health system factors influencing private general dental practitioners (GDPs) engagement in state-funded, contracted primary oral healthcare for low-income adults in Ireland, in which circumstances, for which groups, how and why.

**Methods:**

Nineteen realist interviews were conducted with frontline GDPs, health system actors and academic subject experts from Ireland and elsewhere. Collected data were then transcribed, coded, and analysed to generate context–mechanism–outcome configurations (CMOCs) and develop an overarching realist programme theory to explain causation.

**Results:**

Thirteen individual and abstracted CMOCs were crafted and subsequently consolidated into five high level CMOCs. GDPs’ engagement with state funded care is influenced by a myriad of complex health system contextual factors. These include low political and resource commitment to oral health; cost containment measures characterised by limited and outdated baskets of care and low remuneration; overtly bureaucratic oversight or contract administrative processes; adversarial communications and the absence of consultative mechanisms between the health system and GDPs. Other factors such as oral healthcare ‘market’ dynamics, GDPs’ professional networks and community ties can also influence engagement in state care.

**Conclusion:**

As Ireland looks to reform its primary oral healthcare system to widen population access to care and meet national oral health policy and WHO commitments on oral health, the findings of this study provide health system leaders with evidence to leverage system change and increase or sustain GDPs’ engagement in state care. Leveraging such change has the potential to improve access to care for vulnerable populations and reduce oral health inequalities.

## Introduction

The World Health Organization (WHO) 74^th^ World Health Assembly (WHA) marked a watershed moment in oral health globally. Recognising the public health burden of oral diseases which, despite being largely preventable, affect almost 3.5 billion globally (now almost 3.7 billion), the assembly adopted resolution WHA74.5 which called on UN member states to embed oral health within non-communicable diseases (NCDs) and universal health coverage (UHC) agendas [1]. This historic resolution led to the development of the WHO Global strategy on oral health (2022) [2] and Global oral health action plan (GOHAP) in 2024 [3]. The GOHAP emphasises the need for health systems to adopt a public health approach to tackling oral health inequities that integrates oral health into primary healthcare. Outlined within the GOHAP are a series of action points to assist member states in achieving UHC for oral health by 2030. Regarding workforce, which is one of the six ‘building blocks’ of health systems [4], member states are encouraged to increase and strengthen workforce capacity to deliver essential packages of oral healthcare and ensure this capacity is sustainable and aligned with current and future population needs. In November 2024, member states adopted the Bangkok declaration: “No Health Without Oral Health” at the first global oral health meeting, reaffirming their commitment to the GOHAP and emphasising the need to strengthen health systems through primary care approaches [5].

### Oral healthcare in Ireland and policy landscape

As one of the adoptees of the Bangkok declaration, Ireland is in the early stages of implementing *Smile agus Sláinte (‘Smile and Health’)* - National Oral Health Policy (NOHP) which was published in 2019, preceding the WHA resolution [6]. The primary goals of *Smile agus Sláinte* are to *“provide the supports to enable every individual to achieve their personal best oral health”* and *“reduce oral health inequalities across the population by enabling vulnerable groups to access oral healthcare and improve their oral health”.* The Republic of Ireland (‘Ireland’) is an island nation and European Union (EU) member state located on the north western edge of Europe and has an estimated population of 5.3 million (2024) [7]. Oral healthcare in Ireland is primarily delivered by private primary care providers and funded privately through out-of-pocket payments [8]. There are however three publicly funded primary oral healthcare services currently available, each targeting different populations and described in detail elsewhere [9]. The Oral Healthcare Service (OHS), formerly the ‘Public Dental Service’ (PDS) is operated directly by Ireland’s publicly funded health service provider, the Health Service Executive (HSE) and serves children under 16 and adults with additional needs. The service is delivered by salaried dental teams and aside from urgent care, is ordinarily targeted at children aged 7–8 and 11–12, however coverage and access are limited owing to resource and capacity constraints nationally [10]. State funded primary dental care for adults is largely delivered through contracting arrangements with independent, mainly self-employed private general dental practitioners (GDPs). The HSE’s Dental Treatment Services Scheme (DTSS) principally serves low income, means tested adults holding a medical card (approximately 25% of the population) and offers a limited basket of care annually [10]. The Dental Treatment Benefit Scheme (DTBS) offers a free annual examination and part-subsidised cleaning to adults who have paid sufficient social insurance contributions on their income [10]. The scheme is administered by the Irish Government’s Department of Social Protection (DSP). Both contracted schemes remunerate GDPs on a fee for service (FFS) basis. The current oral healthcare system was informed by the 1994 Dental Health Action Plan (DHAP) [11].

Recognising the fragmented nature of services and substantial gaps in population coverage, namely among young children and vulnerable adults, *Smile agus Sláinte* proposes reform of state funded primary oral healthcare services in Ireland with greater provision of care by private GDP-contractors [6]. The policy aims to contract routine state-funded care for all children (under 16) and vulnerable groups to private GDPs in addition to existing adult populations currently served. A reoriented OHS will deliver episodic care for vulnerable patients on referral from local GDPs. *Smile agus Sláinte* highlights the need for increased access to routine primary oral healthcare for children under six, establishing ‘dental homes’ in general dental practice where prevention is embedded from early childhood. It also proposes to change how GDPs are remunerated, from FFS to bundled care or blended payments.

### Study rationale

To achieve UHC for oral health, the GOHAP asks member states to *“explore how to optimize private oral health care providers’ engagement.... through appropriate contracting and/or reimbursement schemes”.* However, while models of contracting state-funded dental care are long established globally and regarded as an efficient means of creating capacity within health systems, they are voluntary and therefore reliant on private providers opting-in to such contractual arrangements [12]. Recent years have witnessed a decline in the number of private GDPs participating in the HSE DTSS for low-income adults in Ireland [9]. Since 2016, the number of live GDP contracts on the scheme has declined from just over 1,600 in 2016 to just over 800 in 2023, while the number of GDPs submitting claims, and therefore clinically active on the scheme fell from 1,300 in 2016 to just over 800 in 2022 [9]. Both the DTSS and DTBS were subject to substantial government budgetary cuts in 2010 following the global financial crisis [10]. The number and volume of available treatments on both schemes were reduced and fees remained unchanged on the DTSS until 2022 [13]. The decline in participation has garnered significant political attention owing to access difficulties for patients and widening oral health inequities [14]. From a health policy implementation perspective, it also presents a significant workforce planning challenge in engaging GDPs in any new or revised schemes and creating system capacity to deliver services as proposed in *Smile agus Sláinte.*

Challenges surrounding GDP participation in state funded dental services are not unique to Ireland or dentistry. Workforce recruitment and retention challenges in healthcare are often symptomatic of wider health system dysfunction and represent a complex area of inquiry owing to the open nature of health systems with a myriad of interacting components influencing the behaviour of the system to produce both intended and unintended outcomes [15]. Understanding what these components are and how and why they influence the behaviour of the health system and its constituent parts, for example, the workforce can provide policymakers and system leaders with insights and evidence for developing mitigating strategies to leverage system change and increase workforce engagement.

There is a sizeable body of international research examining GDP participation in publicly funded dental services. In our earlier realist review, we developed a range of theories relating to how and why health system factors influence private GDPs participation in state funded contracted care [15]. Key systems’ contexts identified included low political and health system priority for oral healthcare, funding constraints, and change implementation with minimal professional consensus. Where contracts limit GDP decision-making and perceived ability to deliver high quality care this can lead to feelings of lost control, stress, low morale, mistrust of the system and feeling undervalued. Qualitative data which informed our theories primarily came from the UK literature however findings were often reported from the perspectives of GDPs unhappy with contract terms, with health system perspectives largely absent from the data. Furthermore, despite the aforementioned workforce and access challenges, we found an absence of primary, qualitative research examining this topic in the Irish context.

In light of extant challenges in contracted dental care and the absence of primary research in this area, in this study, we collected qualitative data to further test and build upon some of the theories from our realist review in the Irish context and gain a deeper understanding of the current workforce challenges in contracted dental care in Ireland – namely the DTSS for low income adult populations.

## Aim

The aim of this study is to understand what are the health system factors influencing private GDPs engagement in state-funded, contracted primary oral healthcare for low-income adults in Ireland, in which circumstances, for which groups, how and why.

## Materials and methods

### Rationale for adopting a realist approach

For this study, we conducted semi structured, qualitative realist interviews in accordance with an internal team protocol. Ethical approval was granted by the Trinity College Dublin Centre for Health Policy and Management/Centre for Global Health Research Ethics Committee on 30^th^ October 2024 (REAMS No: 1926). Throughout, careful attention was paid to ethical aspects of this study including obtaining informed consent and maintaining confidentiality and participant anonymity.

As with other realist methodologies, this theory-driven approach involves generating, testing and refining theories of how phenomena (or ‘programmes’) work. Specifically, they seek to uncover underlying (hidden) mechanisms (‘M’) which are triggered or modified in specific contexts (‘C’) to produce given outcomes (‘O’) of interest [16]. In realist research, ‘context’ refers not only to physical settings, but a broader set of conditions in which phenomena occur, including individual characteristics, interpersonal relationships, institutional norms, and wider political, socio-economic and cultural settings or circumstances. ‘Mechanisms’ are underlying, often hidden generative processes, such as individuals’ reasoning and responses which are triggered in certain contexts, resulting in outcomes. ‘Outcomes’ are the intended and unintended consequences that arise when mechanisms are activated in specific contexts; these may be proximal or distal and tend to follow semi-regular patterns, though variation is expected given the complexity of social systems. The interplay or causal relationship between these (i.e., C + M = O) are represented by Context-Mechanism-Outcome Configurations (CMOCs) which form the building blocks of realist methodologies [17]. This realist logic of analysis is particularly suited for investigating causation in phenomena operating within complex systems, such as health systems [18].

### Initial programme theory

**Fig 1:**
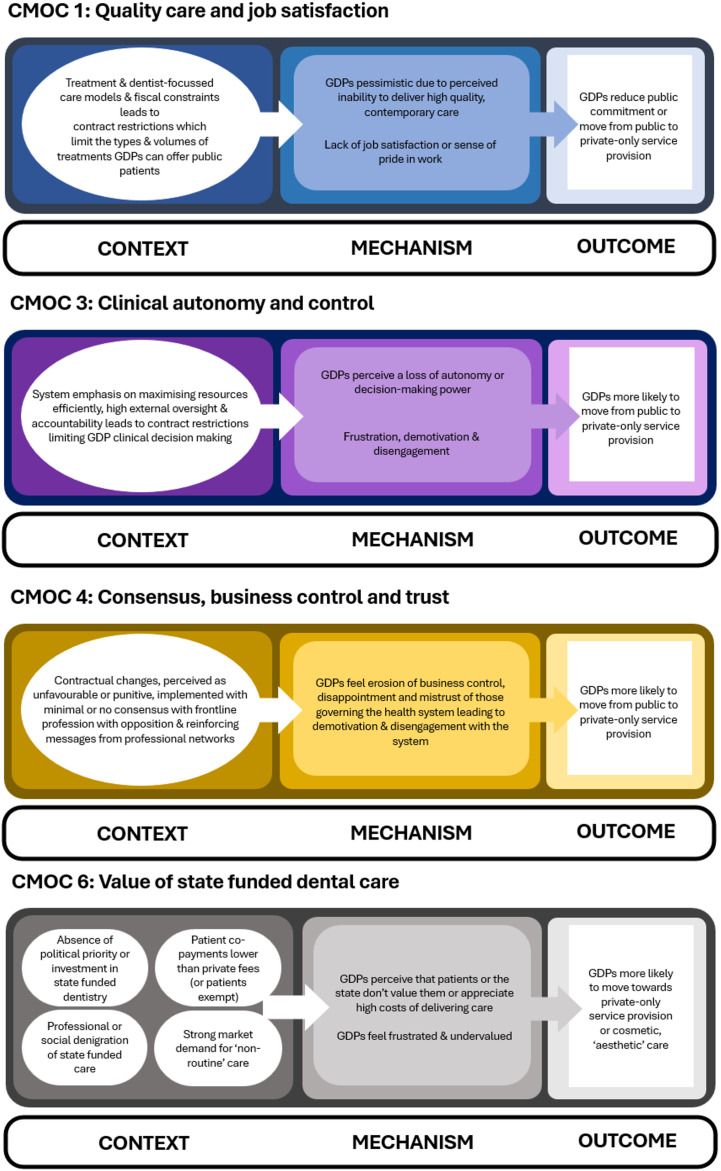
Initial programme theory: CMOCs 1,3,4 and 6 from realist review of the international literature.

This study was informed by an initial programme theory developed from our realist review [15]. Specifically, we utilised four CMOCs (CMOCs 1, 3, 4 and 6) from the review (Fig 1) as the initial focus and starting point for this study [15]. We selected these CMOCs as we felt they had most transferability and relevance to the Irish oral healthcare system based on the structure and governance of contracts and the research team’s prior knowledge of the Irish oral health system. These CMOCs, which had strong evidence base in the existing literature, informed our interview schedule which was developed using realist interview language and followed the RAMESES II Project guidance on realist interviews [19,20]. Consistent with realist logic, the four CMOCs from the realist review represented provisional theories from the international literature which were used as a ‘springboard’ from which to explore how contexts and mechanisms operate within the Irish system. This interview study allowed us to adapt and expand these CMOCs through a process of theory testing and refinement, rather than just theory confirmation. For this study, we wanted to focus on high level health system factors which influence private GDPs’ engagement in HSE-contracted care for low income adults in Ireland (i.e., the Dental Treatment Services Scheme, DTSS) therefore we utilised the WHO health systems building blocks as our framework [4]. This framework comprises six key elements, namely service delivery; workforce; information; medical products, vaccines and technologies, financing and leadership/governance. Additionally, ‘people’ (or communities) are sometimes considered the ‘seventh’ block and one which we considered important in the Irish context as populations expressing a demand for care [21].

### Participant recruitment

A total of 19 interviewees were recruited for this study via purposive and snowball sampling. Participants were recruited between 31^st^ October 2024 and 23^rd^ January 2025. Purposive sampling, sometimes called ‘judgement sampling’ is a recruitment tool, widely adopted in ethnographic and qualitative research where individuals are selected for interview based on their extensive knowledge or experience in the field of inquiry [22]. Health system actors and academic subject experts were identified by members of the research team who have broad networks in oral healthcare in Ireland and via interviewee recommendations and contacted directly via email. A ‘call for participation’ campaign targeting GDPs was circulated on social media and shared through the PI’s professional networks. Prior to recruitment, a sampling frame was developed with participants being recruited based on their significant experience working in or having in-depth knowledge of the primary oral healthcare system in Ireland and their ability to contribute to, refine or consolidate programme theory. As subject diversity is key to informing the development of rounded programme theory, participants from a range of clinical and non-clinical backgrounds were recruited including general dental practice (i.e., practice principals and associates, current and former contractors, urban and rural practising), health service dental governance and management (e.g., contract management, administration, payments), health policymaking and implementation (civil service and HSE), and subject area expertise including dental educators (Table 1). Of the six frontline GDPs recruited, three were current DTSS contract holders, two were former DTSS contractors and one had never held a DTSS contract. GDPs practised across urban, satellite commuter, rural and remote coastal areas.

**Table 1:** Interviewees.

Participant	Number
Frontline GDP practising in Ireland	*6*
Health service manager or other official	*7*
Academic subject expert – Ireland	3
Academic subject expert – international	3
**Total**	**19**

### Data collection

Prior to use, a draft interview schedule was piloted among a small convenience sample (n = 3) of non-dental researchers known to the principal investigator (PI). These were informal, and data captured were not utilised in the analysis. The purpose of piloting was to gauge interviewees’ understanding and comprehension of questions. Participants provided informed and written consent to participate. Following this, semi structured, one to one interviews with all 19 participants were conducted between 15^th^ November 2024 and 7^th^ February 2025. The majority of interviews (n = 17) were conducted online via videoconferencing platforms or by telephone while two were conducted in person. All 19 participants were interviewed once, providing enough data to achieve theoretical sufficiency Two interviewees were later contacted by email and asked to provide additional contextual information relating to information provided in their interviews. Interview duration ranged from 25 to 103 minutes (median = 60 minutes). We utilised a flexible realist interview schedule to guide interviews (supplemental) which was informed by the RAMESES II realist interview guidelines and Manzano et al (2018) [19,23]. Interviews were conducted by the lead author (PL) using a realist technique known as the ‘teacher-learner’ cycle [24]. In interviews, participants were asked a combination of open-ended questions (to enlighten possible new CMOCs) and presented with theories based on selected CMOCs from the earlier realist review. Interviewees were asked to reflect on whether CMOC theories fitted with their own perceptions and experiences in the Irish setting (and in what ways) and to modify or refine these them accordingly. The ‘teacher-learner’ cycle entails the researcher first teaching the interviewee about their theories, with the interviewee then teaching the researcher about their perceptions and experiences [24]. This process helps the researcher to explore how different mechanisms and contexts may result in both intended and unintended outcomes and refine theories. This approach differs to standard qualitative interviews where the interviewer ordinarily puts aside their own preconceptions or assumptions. By their very logic, in realist interviews the interviewer brings their own theories, which has an influence on the types of questions and how they are asked. Similarly, the interviewee will have their own ideas about what the interviewer is interested in, which influences the answers they provide. In theory-driven realist research, the interview is used as a means for empowering participants to revise and expand putative theories. Interviews were recorded and transcribed verbatim using a college approved, encrypted and password protected laptop.

### Coding, analysis and synthesis

Preliminary analysis of interview data took place while interviews were ongoing. This allowed for the updating of the interview schedule and for revised theories to be presented to subsequent interviewees. Interview transcripts were imported and coded in the style described by Papoutsi et al. (2018) using NVivo 12 Plus qualitative analysis software (QSR International, Warrington, UK) [25]. Codes, which were based on what interviewees described, were developed initially using a narrow deductive coding framework from the realist review findings and interview schedules (e.g., ‘funding’, ‘quality care’) but was primarily inductive and retroductive in nature, based on concepts emerging from the interview data. Coding was undertaken by the lead author and PI. Codes were then grouped conceptually by similarity and mapped against the WHO building blocks to understand what issues were emerging from the data and create health systems-focussed categorisations. Coding and categorisation were conducted by the lead author (PL) with a senior supervising co-author (SB) reviewing these independently. These were subsequently discussed with, and sense checked by remaining members of the research team.

The categorised data were then analysed using realist logic by applying labels of ‘context’, ‘mechanism’ or ‘outcome’ to relevant sections of text. By adopting a realist analytical approach, we were able to identify (a) outcomes related to GDPs’ engagement in state funded contracted dental care for low income groups in Ireland, (b) the circumstances in which these outcomes were likely to occur, from a health systems perspective (contexts) and (c) why these outcomes occurred (mechanisms).

Over a period of four months, CMO configurations were constructed iteratively by PL and senior supervising author JF, with input from SB and BD. These were then utilised to inform our overarching programme theory. The process of coding data, building, iteratively refining and crafting CMOCs took place through early and often sharing of emerging contexts, mechanisms and outcomes using a series of online shared documents (Microsoft Word and PowerPoint for data visualisation), and through regular meetings where CMOCs were discussed and refined. These processes, built in to the research served as a means of quality assurance were not quantitative, but rather iterative and dynamic in nature. This process resulted in 13 individual CMOCs presented in abstracted format. Thereafter, these were consolidated into 5 CMOCs specific to the Irish context and based around the broad themes or concepts under which these were felt to fall under by the research team.

### Project advisory group

In line with other realist methodologies, a project advisory group (PAG) workshop was convened to consider emerging findings of this study [26]. Workshop attendees included two members of the immediate research team (PL and SB), oral health services, workforce and policy researchers (n = 2), population health/health systems researcher (n = 1), frontline GDP with union experience (n = 1), civil servant working within the Department of Health (Ireland) Oral Health Unit (n = 1) and patient/public representative (n = 1). An online workshop was held in June 2025 during which emerging theories were presented to stakeholders. PAG members were given the opportunity to confirm, refine, or refute these, based on their prior knowledge and experience.

### Reflexivity

A reflexive diary was maintained by the lead author after interviews. This facilitated reflective awareness of potential internal and external influences on data interpretation, including professional roles and the ethical implications of being a clinician-researcher. To support objectivity and minimise bias, the data were shared early and often with, and reviewed independently by senior supervising co-authors throughout the analysis and synthesis.

## Results

Utilising data from interviews (supplemental file), we crafted 13 individual CMOCs, outlined in Table 2 below. These are presented as ‘middle range theories’ which means they are presented at a level of abstraction that is specific enough to clearly explain the phenomenon, but they are close enough to observed data to be incorporated in propositions that permit empirical testing [27]. Thereafter we present five consolidated CMOCs providing detailed narratives of the CMOCs in action in the Irish context.

### Abstracted Context-mechanism-outcomes configurations

Individual, abstracted CMOCs and illustrative data are presented in Table 2 below.

**Table 2:** Individual context-mechanism-outcome configurations and illustrative data.

CMO configuration	Illustrative quotes
**CMOC 1: Political and resource commitment to oral health and system fragmentation**	
***CMOC 1*** When oral health holds limited political capital with broader financial constraints, and politicians perceive GDPs as financially privileged or opportunistic **(C)**, health system leaders or managers deprioritise oral health within broader health service planning triggering feelings of irrelevance, lack of integration across health services and lack of support (e.g., clinical and non-clinical supports) **(M)**. This leads to a mismatch between the articulated need from GDPs and service provision **(O/C)** and in turn, this results in loss of trust in the public system among GDPs and disengagement with strategic reform **(M**) leading to contract resignations in favour of wholly private practice **(O)**.	*“There’s no like, I haven’t had a single support on that. I haven’t found support on how to deal with complex patients. If I’m not able to treat them in practice, where do I refer them? Like, I would have some patients coming in, and they’d be disabled, you know, physically disabled or mentally impaired, and I wouldn’t know where to refer them, except to the dental hospital, just because I was [a student] in the dental hospital, but you don’t have that specific guidance on what to do with these kind of patients, support, talking to someone - no, the support isn’t there really. It’s kind of… they give you the booklets, they give you the prescription pads, they tell you need to send for approval....and, you know..... good luck…” – Interviewee 13*
**CMOC 2: Financing and oral health market dynamics**	
***CMOC 2.1*** When political pressures constrain funding causing limits to remuneration amidst rising operational costs **(C)**, GDP contractors perceive the current model as unsustainable and feel undervalued by the state **(M)**, leading to disengagement from public schemes and movement towards private-only care **(O)**. ***CMOC 2.2*** When there are strong markets for private and aesthetic treatments, combined with professional and societal importance and emphasis on cosmetic treatments and the absence of state system mentorship **(C)**, GDPs perceive private practice as more lucrative and less demanding compared to public schemes **(M)**, particularly among early-career GDPs. This prompts GDP non-engagement or disengagement from public schemes in favour of private practice **(O)**. ***CMOC 2.3*** In socioeconomically disadvantaged or rural areas with weaker private markets and when GDPs have strong community ties **(C)**, GDPs feel a moral obligation to provide public care and are more accepting of income and scheme limitations **(M)** leading to increased willingness to remain in public care, adapting their public commitment in order to maintain a capacity for supplementary private work using strategies such as limiting public patient intake or geographical ring-fencing **(O)**.	*“There is absolutely no funding at all for any part of it, and you carry all of the costs and all of the risks. And you know, if, if you have a practice with quite a, you know, a big base of medical card patients, you’re carrying a big risk, then when the scheme is changed without consultation, you know you’re carrying the brunt of that cost anyway, and then you still have to pay the cost, whether or not you’re getting sufficient payment for the treatments that you’re carrying out. So it’s basically a cost benefit analysis. Can you cover your costs? And can you pay your staff? That’s a big thing. And can you make a living yourself that you could raise a family on? And that’s you know, anyone who runs a business, you have to, you have to cover your costs. Otherwise, you know, there’s no point.”- Interviewee 19* *“I think the graduate coming out now, there’s no real effective mentorship that I can see like. They’re coming into corporate practice and the dollar rules. They’ve little or no mentorship within practices and it’s all associate-driven like and it’s money, money, money”- Interviewee 17* *“I would have left the scheme if I could, but I can’t, because of my location....I can’t leave the scheme, so I’m not Mother Theresa but I’m kinda, I’m trying to……reflect….on…..you know….and it’s the hard cases..they’re coming down, they cant… I mean, it would be tragic if these people didn’t have access locally…. to the scheme. And… you know…. it is…. it is satisfying, more satisfying than if you just did your private work…its just going, just going wow, can we operate the scheme… that worked very smoothly for that person…that’s another happy customer.” – Interviewee 16*
**CMOC 3: Contract administration**	
***CMOC 3.1*** When cost containment limits treatment options and increases the likelihood of difficult conversations between GDPs and patients **(C)**, GDPs become frustrated at the inability to provide comprehensive care and feel they are treating patients inequitably **(M)** leading some GDPs to remain in public care to advocate for, and serve those they can **(O)**. ***CMOC 3.2*** When public services face financial constraints limiting treatment options, GDPs need to have difficult conversations with patients **(C)**. GDPs experience professional dissonance and frustration due to compromised clinical decision-making and difficult patient interactions regarding costs compounding low morale **(M)**. In turn this leads to GDPs being more likely to reduce or fully withdraw from participation in public schemes in favour of private practice **(O)**. ***CMOC 3.3*** When prior approval processes lack clear guidance and standardisation **(C)**, there is variability in their interpretation and implementation **(M)** leading to restricted clinical decision making among GDPs **(O/C)**. In turn this leads to perceptions of micro-management, perceived mistrust of practitioners on the part of managers and increases tension between GDPs and managers **(M)** which makes GDPs avoid the approval process and increases the likelihood of leaving the public system **(O)**. ***CMOC 3.4*** When prior approval processes lack clear guidance and standardisation **(C)**, there is variability in their interpretation and implementation **(M)**, leading to GDPs having conversations which disappoint patients due to unsuccessful approval outcomes **(O/C)**. This increases GDPs feelings of guilt, discomfort, and perceived systemic unfairness **(M)**, makes GDPs avoid the approval process and increases the likelihood of leaving the public system **(O)**. ***CMOC 3.5*** When prior approval processes lack clear guidance and standardisation **(C)**, there is variability in their interpretation and implementation **(M)**. If approval is successful, validating GDPs clinical decisions **(O/C)**, GDPs feel supported with improved relationships with management and increased willingness to accept limitations of the scheme **(M)** leading to an increased likelihood of staying in schemes **(O)**.	*“I certainly went into dentistry to try and A) do the right thing, and B) help people. And if, if you’re hampered in your ability to do that, that’s very difficult…. it’s extremely challenging not to be able to give the patients the care that A) you want and B) they need, particularly when patients can’t afford it. That’s one of the reasons I’ve continued with the medical card scheme.” – Interviewee 19* *“the fact that they [DTSS contractors] cannot treat patients the same and provide the same level of treatment, I mean, even down to the fact they’re limited to a certain number of fillings, so people come in and they could have a mouth full of decay in that socio-economic group, and you’re ending up picking the two fillings you can do, and then whatever happens after that is between the dentist and the patient. So it’s a sense of utter frustration I think people have, and it’s overcome any sense of altruism and or, you know, sort of moral obligation to treat those ‘well less off’ which I do believe exists in in the profession” – Interviewee 4* *“I suppose I feel that I’m not being trusted to make adequate clinical decisions. If I feel that a patient, say, for example, and this happens very regularly, a tooth breaks, a portion of the tooth comes away, and you fill the tooth. Couple of years later, another portion comes away, and then you have to get approval to refill that tooth. The tooth needs filling! If you don’t fill it at some point, it’s going to need extracting. And the longer it’s left, the greater the risk of that. And that is quite frustrating, because the simple solution will save a lot of trouble. But it’s a question…. it feels like your clinical decisions are being questioned by somebody who hasn’t seen a patient. And it also… I find… I feel like my honesty is being called into question. I really do……yeah, it’s as simple as that” – Interviewee 19* *“someone ‘up there’ is saying, ‘no’… they’re not approving, you know, and like, it leaves it in your hands to deliver that news to the patient. It’s unfair, because you know the patient needs the treatment and they’re not getting it, and it’s unfair on the dentist to be like, ‘oh I’m so sorry you weren’t approved for the treatment, you have to pay X amount of money to get it on the private scheme’. You know, it’s really annoying” – Interviewee 13* *“I am inclined to ensure that within what they are eligible to provide, that there is as little restriction or limitation or hindrance on our behalf put on those dentists as possible. Because, you know, we are trying to keep the last man, you know, the last man standing, in effect. But you know, the HSE aren’t stupid, the Principals know that we’re at the mercy of the dentists. And so I would be very surprised if, if you looked nationally, although there may be variability, I think there will certainly be a lot of, kind of, really serious efforts made to try and keep this moving as quickly as possible, because otherwise it falls back on the HSE.” – Interviewee 5*
**CMOC 4: Quality assurance (probity)**	
***CMOC 4*** In the absence of robust, standardised quality assurance mechanisms, combined with perceived accusatory or confrontational communications from health service management **(C)**, GDPs experience feelings of unfairness, mistreatment and defensiveness **(M)**. Such experiences reinforce conflicting relationships, prompting GDPs to disengage or withdraw entirely from public schemes, thereby exacerbating professional isolation and mistrust **(O)**.	*“Yeah I mean, they are certainly dictatorial. I mean, I had, many years ago, a dispute over the number of surgical [extractions] I was doing. I do take out multi rooted teeth, in general I section them- I have VarioSurg with saline irrigation. I take them out as sections, pretty much every multi-root [tooth]. And I have no issues, but you know that, they have all their metrics in [HSE] ‘central’, wherever it is. So that did trigger a bit of probity. I felt that it was way over the top.” – Interviewee 16*
**CMOC 5: Communication and engagement**	
***CMOC 5.1*** When collective contract negotiations between the health system and GDP representative bodies become unilateral, rigid, and adversarial **(C)** this leads to entrenched positions and mutual suspicion, defensiveness and negative interpretations of communications **(M)** and subsequently reduces GDPs willingness to engage in state-funded care and health care managers less willing to openly engage or negotiate with GDPs **(O)**. ***CMOC 5.2*** In contexts lacking formal feedback and engagement mechanisms during health system changes without accurate consensus on the outcome **(C)**, GDPs feel ignored, isolated, and excluded from decision-making processes **(M)** leading to decreased engagement with health service management and state-funded care systems **(O)**. ***CMOC 5.3*** When GDPs have open communication channels and formal feedback mechanisms within the health system **(C)**, GDPs feel understood, supported, and valued by health system management **(M)** leading to more positive relationships, reciprocity, and increased sustained engagement by GDPs in public scheme **(O)**.	*“I changed a referral letter - the content, there was a kind of a referral template for them to refer in oral surgery referrals. And somebody, anyway, one of the GDPs got upset about this and the next thing, there was this huge backlash! And I wasn’t saying we weren’t going to take all oral surgery referrals….I might have made some kind of reference to there needs to be, that the impacted wisdom teeth needed to be referred in as per the NICE guidelines. But anyway….the point was, the reaction I got was so negative that I thought, well you know what, I’m never meeting with these guys” – Interviewee 9* *“That [abolition of local monitoring committees] removed the local input, if you like, it became, then more of a kind of ‘us and them’ thing really, then you know, where you had the payments and the politicians and then and the policy makers sitting with the professional representative groups. And I think the people further down the chain felt more removed. They just didn’t know what was going on. And you know, over time, there was a lot of lot of arguments and a lot of lot of misinformation that went out, but more and more people started leaving the scheme because they just couldn’t, they just said, ‘No, I don’t want to be dealing with the HSE’.”- Interviewee 3* *“I actually found the relationship, I could pick up the phone and have the chat [with the HSE dental manager], which was a big help - having a conversation, a direct conversation by phone helps enormously for everyone to know exactly where everybody else is up to. Yeah, I would be a great believer in picking up the phone and having a chat.”- Interviewee 19*

**C** = context; **M** = mechanism; **O** = Outcome; **O/C** = outcome functioning as context; **GDP** = general dental practitioner; **HSE** = Health Service Executive; **DTSS** = Dental treatment Services Scheme

### Consolidated Context-mechanism-outcomes configurations

#### Consolidated CMOC 1: Political and resource commitment to oral health and system fragmentation.

**Fig 2:**
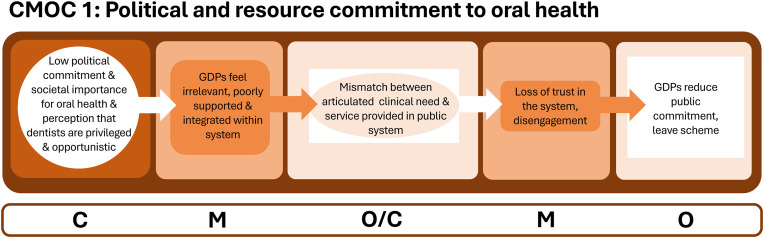
Consolidated CMOC 1: Political and resource commitment.

CMOC 1 (Fig 2) describes the impact of a historical absence of, and low priority and commitment to primary oral healthcare in Ireland on the deprioritisation of services, health service delivery and system fragmentation wherein GDPs are poorly integrated into the public healthcare system. This exists within a broader context of dentistry yielding limited political capital and perceptions among dentists that oral health holds limited population importance, and system change inertia in Ireland. Furthermore, interviewees perceived that individuals within positions of power, for example government ministers historically may have held negative views of GDPs as opportunistic, further contributing to low priority for oral health.

The deprioritisation of oral health in Ireland triggered in the context outlined above was notable in 2011 following the great recession when State schemes were scaled back, and fees were frozen as part of Irish Government austerity measures in the form of disproportionate oral health budgetary cuts. For GDPs holding these contracts, this led to a perception that they were irrelevant to the Government, and a sense of denigration (as well as shock, fear of financial hardship and uncertainty). This sense of irrelevance and degradation was also reported by GDPs in 2020 when government promises to provide personal protective equipment (PPE) to DTSS contractors (in the wake of COVID-19) did not materialise despite calls for this (and the introduction of other supports) from those within health service dental management. GDPs also reported feeling unsupported and a sense of abandonment and isolation within the health system, perceiving that the government did not recognise or understand their plight.

More broadly, interviewees perceived GDPs as being poorly integrated within the Irish general and oral healthcare public systems. A notable manifestation of this fragmentation is the absence of, or inconsistencies in availability of clinical and non-clinical supports for state-contracted GDPs, for example specialist referral pathways or scheme training and a perceived absence of collaborative working relationships between contracted GDPs and local community dental services.

These outcomes function as additional contexts triggering both individual and collective loss of trust in the ‘state’ and health system by GDPs (notably following the deprioritisation during the austerity and COVID-19 periods), demotivation to provide state-financed care and disengagement with HSE-contracted care. The absence of supports has triggered feelings of being unsupported by and isolated within the health system. Collectively these mechanisms have led GDPs to reduce their public commitment and have led some to leave the scheme altogether.

#### Consolidated CMOC 2: Financing and oral health market dynamics.

**Fig 3:**
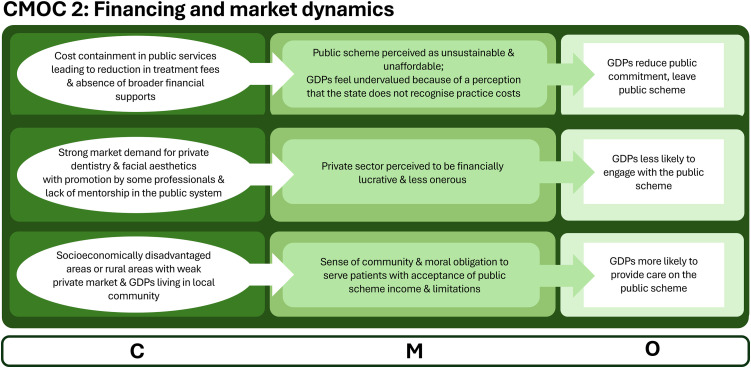
Consolidated CMOC 2: Financing and market dynamics.

CMOC 2 (Fig 3) explains the role of health system financing and the wider dental care ‘market’ in Ireland on GDPs’ engagement in HSE-contracted care. Building on some of the same contextual factors in CMOC 1 – austerity measures, cuts to state schemes, constrained fees and the absence of practice or additional financial supports (e.g., domiciliary care payments), interviewees reported that for some GDPs these factors, alongside inflation and rising practice costs, can trigger perceptions that the state or health system does not recognise the costs of delivering primary dental care and feelings of not being valued or rewarded appropriately for their efforts in providing state funded care. Where GDPs (primarily practice owners carrying business costs and risks) come under significant financial pressure, this can result in anxiety, and a sense of unsustainability and unaffordability in continuing on public schemes. Cumulatively, these factors can lead to GDP demotivation to provide state-funded care and disengagement with HSE-contracted care.

Concurrently, wider economic conditions and the private market play a significant role in influencing GDP participation in state contracted care – especially amongst urban and early career practitioners. Where there is a strong population demand for private dental care and medical (facial) aesthetics, this can trigger perceptions that this sector is financially more lucrative and less onerous compared to the DTSS whose patient cohort may be regarded as more complex and challenging to treat. Interviewees at both frontline and health systems level expressed views that a profit-focused, marketing culture in private dentistry had emerged in Ireland where the wider dental profession including industry, corporate bodies and professional representatives were highly influential in defining ‘oral healthcare’, promoting aesthetic treatments and independent practice and that this was buoyed by high societal interest and demand in cosmetic care. Some also perceived that there was an absence of state-supported system mentorship or a culture that engenders a sense of public service ethos and social accountability among dentists. The effect of the above on GDP behaviour is the non-engagement of GDPs, notably early career dentists in HSE-contracted care in the first instance, as well as disengagement among existing contractors.

Conversely, where there is a less buoyant market in private oral healthcare, for example during recessionary periods, in socio-economically disadvantaged areas or rural areas where a high proportion of the local population is eligible for publicly funded care, GDPs may be more likely to provide state funded care, because they perceive this to be an acceptable means of earning an income and therefore may be more willing to accept scheme shortcomings as a result. Similarly, when mid or late career GDPs’ practices are located in rural areas and where GDPs reside in and have personal ties with their local communities, they may be less likely to leave the scheme, because they feel a sense of community and a moral obligation to serve their patients. Some GDP interviewees in these areas reported that they had chosen to remain in the DTSS but adapted their public commitment in order to maintain some capacity for private work, for example by ‘ring-fencing’ their locality as a *de facto* ‘catchment area’ or closing their books to new DTSS patients. Some GDPs reported that they derived a sense of job satisfaction in providing care to vulnerable groups.

#### Consolidated CMOC 3: Contract administration.

**Fig 4:**
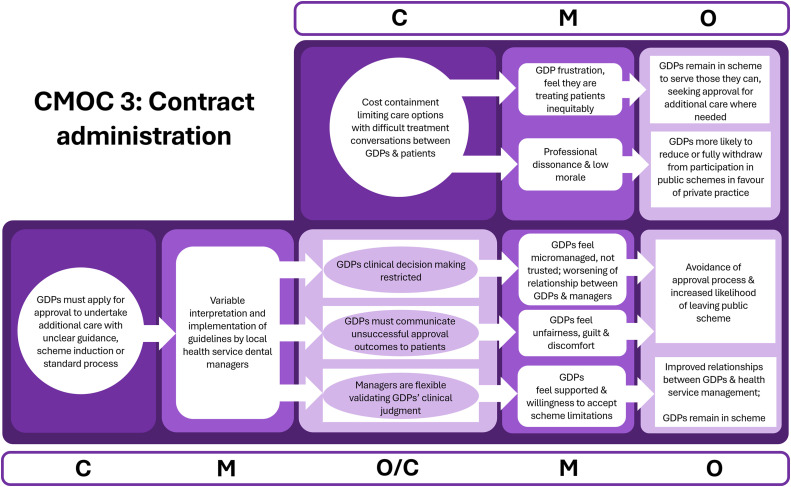
Consolidated CMOC 3: Contract administration.

The third CMOC explores the impact of scheme administration and oversight both at national and local levels in influencing relationships between GDP contractors and the health system, and engagement in care (Fig 4). As highlighted in CMOCs 1 and 2, cost containment measures introduced in 2010 limited the number of restorative and preventative treatments available to HSE-contracted GDPs. Notably, these contract changes allowed for ‘unlimited’ extractions without the need for prior approval. More broadly, interviewees expressed views that the current contract was outdated and lacked intent in terms of clearly defining desired population oral health outcomes. For some GDPs (especially those with a strong sense of social accountability), operating under these contract limitations can trigger feelings of frustration because of a perceived inability to offer a full complement of necessary care they want to on the scheme and perceptions of treating their patients inequitably. Notably for these GDPs, this has led some to remain in the scheme and to try and serve those they can including advocating for their patients to receive more care through prior approval.

Similarly, interviewees spoke of difficulties in clinical decision making, namely which teeth to restore and which to extract or leave actively diseased and of discomfort in having conversations with patients on alternative private treatments and costs. They reported feelings of frustration, professional dissonance and low morale at having to make difficult decisions which they perceived not to be in their patients’ best interests and discomfort because their patients may not be able to afford private care. This can lead some GDPs to reduce their public commitment or leave the scheme in favour of fully private care.

The process by which GDPs must apply to local HSE dental managers for approval to undertake additional treatments (‘prior approval’) and its governance was a factor frequently cited as having a major influence on relationships and GDP engagement with state care. From a scheme governance perspective, a view held among many interviewees working at health systems level was that there is a lack of clear guidance and standardisation of the prior approval process or accountability within health service management hierarchy. There were perceptions that local health service dental managers may have differing levels of interest, experience in or availability to manage approvals (due to other commitments) and that some may be overly dogmatic in their interpretation of contract rules, for example defining patient vulnerabilities. These factors trigger differing approval approaches and outcomes nationally and interviewees opined that greater standardisation and calibration or consensus among managers was required, for example on ‘borderline’ approval cases.

GDPs widely reported feeling frustrated at having to apply for prior approval in the first instance because of a perceived loss of clinical decision-making power and a sense of being ‘micro-managed’ by health system managers. The prior approval process (and other aspects of the scheme, for example seeking payment) was widely regarded has having a high degree of administrative burden on both GDPs and health service managers, heightening frustration. When prior approval requests are rejected by dental managers, this triggers (or reinforces) perceptions of lost control and micromanagement. Some also reported feeling like their professionalism and honesty were being questioned and that they were not being trusted to make appropriate clinical decisions.

Amid an absence of GDP scheme induction or training and publicly available information on, and perceived ‘grey areas’ surrounding scheme entitlements and eligibility, GDPs are often tasked with ‘*breaking the bad news’* on prior approval rejections to frustrated patients. Interviewees reported this triggered perceptions of system unfairness among contractors, guilt and further frustration and discomfort in having to provide (often time consuming) explanations of system limitations and discussions on out-of-pocket alternative costs.

The resulting outcomes of these scenarios are strained relationships between practitioners and health system managers, GDPs losing motivation to deliver public care and disengagement from the DTSS or as some suggested, GDPs persisting on the scheme but limiting their engagement to provision of ‘above the line’ treatments without approval only and ultimately limiting their engagement with health system management.

Conversely when prior approval requests are readily approved by health system managers who may exercise a high degree of local flexibility in their interpretation of contract rules (in an effort to keep GDPs in the scheme), this can reduce GDP frustration and engender a sense of being supported, control and ability to accept scheme shortcomings. Many felt that this did, and had the potential to, improve working relations between GDPs and local management and keep GDPs engaged in the state sponsored care.

#### Consolidated CMOC 4: Quality assurance (probity).

**Fig 5:**
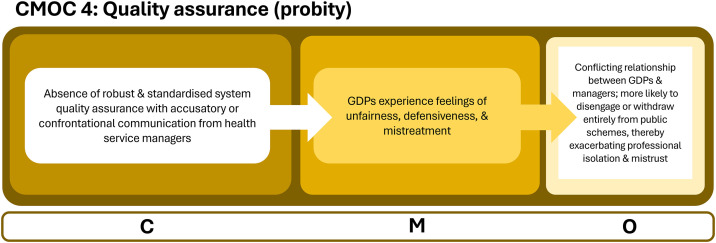
Consolidated CMOC 4: Quality assurance (probity).

CMOC 4 examines quality assurance, referred to as ‘probity’ in the Irish health system (Fig 5). Like prior approvals, contexts and mechanisms relating to probity emerged as those frequently considered key in informing relationships and GDP attitudes towards, and participation in state funded care.

Contextually, many expressed the view that GDPs in Ireland are underexamined with insufficient checks in place to ensure GDPs are adhering to contract rules, namely claiming appropriately, and that there were insufficient clear clinical standards guiding GDPs or health system managers. There was a view that historically, some GDPs had engaged in opportunistic behaviour and that this may have contributed to spiralling scheme costs (prior to austerity cuts) and a dim political view of GDPs among some in power. Many interviewees also suggested that there was a background culture of opportunistic and evasive behaviour (*‘ducking and diving’*) in Ireland and an absence of a culture of continuous improvement or system oversight among GDPs.

Interviewees frequently discussed historical health system approaches to probity and missed opportunities to develop robust quality assurance which have been perceived negatively by both GDPs and health system actors and contributed to a lack of system ‘buy in’ among GDPs. One such context being a ‘blanket’ rather than targeted approach to probity – namely with respect to GDP contractors being asked to make self-declarations and repay on over-claimed complex extractions. At health system level, some suggested that suboptimal claims data validation contributed to blanket approaches to probity.

Among GDPs, this triggered a range of mechanisms. For some, the immediate response was upset and panic (with some reportedly making overpayments to the health service) and a perception that they had done something wrong. For others, notably GDPs who were committed to the scheme or who had not engaged in opportunistic behaviour, this scenario trigger feelings of unfair and disproportionate treatment by the HSE.

The way in which health system management communicates with GDPs on probity (and other issues such as patient complaints) and the language adopted was perceived by GDPs (and indeed some within HSE management) as unfriendly, confrontational and accusatory, which contributed to feelings of wrongdoing among dentists, mistrust in and suspicion of the health system among GDPs.

There were expressions of disappointment among interviewees that a peer-led probity scheme did not reach maturity or see widespread implementation. The initiative saw GDPs undertaking checks on each other’s patients and was perceived as a positive, collegiate process among GDPs and one which facilitated GDP system ‘buy in’.

The contexts and mechanisms above broadly appear to have resulted in broad GDP dissatisfaction and disengagement from the scheme with some leaving as a result, however for some who choose to remain in the scheme, they reported underclaiming on certain treatments to avoid triggering investigations (even if these were performed legitimately). More broadly these factors have contributed to a system that is widely perceived as adversarial and lacking in symbiosis or collegiality between health service management and frontline GDPs.

#### Consolidated CMOC 5: Communication and engagement.

**Fig 6:**
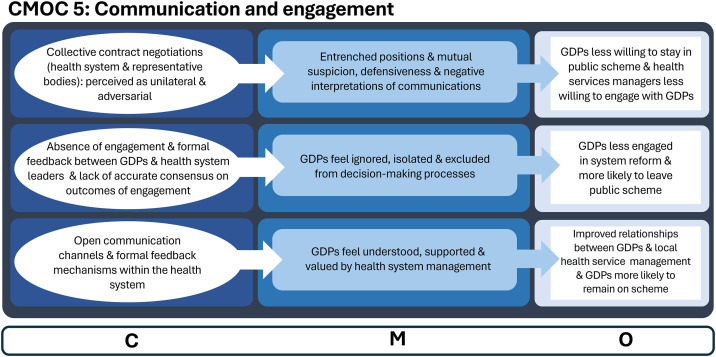
Consolidated CMOC 5: Communication and engagement.

The final CMOC (Fig 6) investigates multiple contexts centred around how the health system and profession engage and communicate with one another. At its highest level, there were reports of fractious collective contract negotiations taking place between the health system and professional representatives which were perceived as unilateral, dictatorial and inflexible by and on both ‘sides’. This context has triggered a mechanism of polarisation and entrenchment of established positions, leading to suspicion and ultimately a loss of trust and an absence of symbiosis between the system and profession. A notable manifestation of this was the collapse of a formal consultative forum between the Irish health system and dentists, which was established to maintain stable contractual relations.

Like CMOC 4, interviewees working within health service management reported that while some written communications with GDPs may be overtly dictatorial and accusatory in tone, they felt there was a propensity for GDPs to perceive any health service correspondence as unfriendly, accusatory or confrontational (irrespective of the actual intent) and that receipt of these communications had the effect of triggering a mechanism of defensiveness and outcry among many GDPs. This had resulted in health service management feeling less willing to engage with GDPs for fear of backlash. They recognised that this had contributed to, and was reinforced by, an adversarial culture and loss of trust and had resulted in GDP disengaging with the health system and leaving the state scheme.

More widely, interviewees perceived that there was an absence of effective consensus and feedback mechanisms or open channels of communication between frontline GDPs and the health system. On system change and governance, there was a perception of a lack of meaningful engagement between the health system and GDPs, and that decisions were often made unilaterally and without consensus or communication (or with minimal engagement only). These contexts have had the effect of triggering perceptions among GDPs that they are ignored by the health system, not involved in decision making processes and ultimately that they are isolated and not part of the system. Interviewees on both sides cited the abolition of local monitoring committees which were forums for GDPs and health service officials to engage with one another and discuss local service operations, as instrumental in creating distance between frontline GDPs and the system, increasing their sense of isolation. Conversely, some working within health service dental management perceived that some GDPs themselves were not willing to engage in conversations on system change or ‘ownership’. The above contexts and mechanisms have led to reduced GDP system ‘buy in’, increased demotivation and disengagement with state dental care, with some reducing their commitment or resigning their contacts in favour of wholly private care provision. In contrast, both health system actors and GDPs reported that when there are open channels of communication and collaborative working arrangements between local health service management and GDPs, this triggered perceptions among GDPs that they were listened to, understood and supported, leading to sustained engagement in state funded care and improved working relationships between practitioners and local health service management.

#### Overarching programme theory.

**Fig 7:**
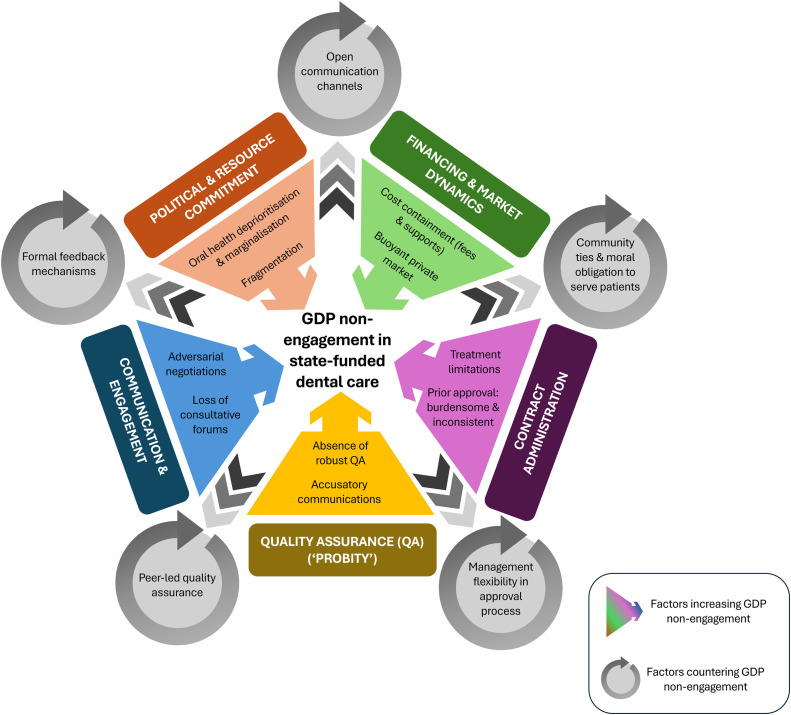
Overarching programme theory.

Informed by the CMOCs outlined above, our overarching, final programme theory provides a full explanation of the study’s findings. This research found that GDPs’ engagement with state funded dental care in Ireland is informed by a myriad of complex and interacting health system factors. These include low political and resource commitment to oral health, financial incentives which are perceived as unfavourable and deficits in system governance, administration and communication. These dynamics have led to professional dissatisfaction and dissonance among GDPs, mistrust of the system, and disengagement from state funded care however the influence of various systems and local level factors can be seen to act as countering forces to such disengagement (Fig 7).

When oral health is deprioritised politically and marginalised within the health system, and amidst historic challenging financial circumstances, this can lead to fragmentation of services and contracts which are designed around cost containment with minimal service or financial supports available to contractors. Against a backdrop of rising business costs, when contracts limit the treatment options available to GDPs, this can contribute to financial and professional burdens. These conditions can result in perceptions among GDPs that dentistry is politically undervalued, and that state funded care is unsustainable, particularly when juxtaposed against a more lucrative (and autonomous) private dental market driven by professional and societal emphasis on cosmetic treatments and absence of state supported system mentorship. These system dynamics can reduce the attractiveness of state schemes, most notably among early-career or urban-practising GDPs. Conversely, economic imperative, community ties and moral obligation to service public patients can sustain GDPs’ participation in state contracted care.

Compounding GDP dissatisfaction are contract governance processes such as prior approval and probity. While these seek to provide contract oversight and accountability, there can be a lack of clarity and inconsistencies in how these are operationalised by health system managers. Coupled with the content and perceived tone of health service communications, GDPs may perceive these processes as overly bureaucratic and accusatory, undermining their sense of clinical autonomy. This can erode professional morale, reduce trust, and lead to defensiveness, strained relationships between GDPs and health system managers and disengagement from public schemes. Management flexibility and GDP involvement with these governance processes have the potential to increase GDP ‘buy-in’.

Finally, efforts at engagement and consensus building through formal negotiations have been mired in perceived unilateralism and inflexibility on both sides resulting in entrenchment of positions, a breakdown in dialogue and the loss of mutual trust. The loss of local consultative structures has diminished opportunities for feedback and ongoing collaboration, leaving GDPs feeling voiceless, isolated, and less willing to engage with the state health system. Where formal and informal structures are in place that facilitate open communication, feedback and consensus building between the health system and GDPs, these can further act as counterbalancing forces and promote GDPs’ engagement.

## Discussion

This realist interview study expands on an earlier realist review of the international literature by the same authors and explains how health system-related contextual factors and hidden mechanisms interact to produce outcomes related to private GDP engagement in state funded, contracted primary oral healthcare for low-income adults in Ireland [15]. This research comes at a crucial time when the Irish health system is experiencing a reduction in GDP engagement in HSE-funded care and against a new policy framework wherein it is anticipated that GDP contractors will provide a greater volume of care on behalf of the state, including care for children [6,9].

As Ireland looks to strengthen its oral healthcare system in anticipation of the National Oral Health Policy’s implementation and meet its international obligations on achieving UHC for oral health by 2030, this study, for the first time, provides robust, primary evidence for system leaders and managers to identify points of leverage for system change and optimise GDP-contractors’ engagement in state care. As highlighted in our earlier review and by others, engaging private GDPs in publicly funded primary oral health as third-party contractors remains a global challenge and is therefore not unique to Ireland [15,28,29]. This is important in the context of the WHO GOHAP which implores member states, including Ireland, to increase workforce capacity to deliver essential packages of care and *“ensure that investment in human resources for oral health is efficient, sustainable and aligned with the current and future needs of the population”* [3]. While the GOHAP highlights the need for workforce expansion through for example, updated scopes of practice, and greater use of task shifting or mid-level practitioners, it recognises the importance of private dental providers and asks member states to explore ways to optimise their engagement in contracted schemes.

From a health systems perspective, the existing evidence pool on this topic in Ireland is very shallow. To date there have been reports and policy papers published by the Irish Dental Association which include excerpts from GDP members’ surveys highlighting many of the same challenges surrounding engagement in the DTSS as uncovered in our study. These include austerity cuts to scheme funding and clinical constraints within contracts [30,31]. While these publications provide useful summaries of the extant problems with state schemes in Ireland from the perspective of GDPs, they do not provide an in-depth appraisal of health system level contextual factors at play or a level of data richness that begins to explain the relationships or causal patterns between these contexts and subsequent GDP (or indeed system) behaviour. From a high level health system and policy perspective, the findings of our study echo those of McAuliffe et al [10]. Their health policy-focussed case study which was also informed by elite interviews with Irish oral health actors, found longstanding deficiencies in primary oral healthcare political commitment, planning and resourcing in the 27 years following the publication of the Dental Health Action Plan in 1994. In particular, it highlights the impact of national level political and health service management ‘disinterest’ in public oral healthcare and funding cuts on the resourcing and operation of contracted schemes. Interviewees reported that they felt that the state had ‘abandoned’ GDP-contractors, leading to the decimation of schemes with some describing the DTSS as ‘medieval’ in its tooth extraction-driven approach, mirroring the sentiments of interviewees in our study.

From a scheme governance perspective, there have been longstanding concerns regarding the absence of robust quality (probity) assurance, accountability and standards in the Irish oral healthcare system. Commencing in 2002, a series of reports commissioned by the Department of Health highlighted deficiencies in probity assurance approaches and estimated that at one time, approximately 10% of DTSS claims were likely to be inappropriate [32]. Despite some progress being made in the mid-2000s, a 2009 report concluded that the QA system was unfit for purpose and positive aspects that had been developed, for example the peer-led examining dentist scheme, had collapsed, exposing the system to significant risk.

More broadly, the findings of this study align with those of our earlier realist review, reinforcing the international literature but with some notable differences which are most likely attributable to system or contract variances. Common findings which informed our original CMOCs and which we have expanded upon in this study include low priority and funding for oral health, system change without consensus and contracts restricting GDP clinical decision-making leading to feelings of lost control, mistrust, and being undervalued by the health system. Another prominent finding from the earlier realist review relates to GDPs’ perceived inability to deliver high quality care on state dental contracts – specifically on the National Health Service (NHS) General Dental Services (GDS) contract in England. The pressure of reaching clinical targets (‘Units of Dental Activity’) set by NHS commissioners, coupled with perceived low remuneration had resulted in GDPs feeling like they were unable to deliver a high standard of clinical care to their NHS patients leading to professional burnout [15]. In contrast, in the present study we found that while GDPs lamented the limited (and perceived outdated) basket of care available on Irish schemes, they did not report that they were hampered in their ability to deliver a high standard of clinical care to their public patients. This could be attributable to the smaller basket of restorative care available on Irish state dental schemes and therefore less ‘scope’ for GDPs to deliver care which they perceive to be sub-standard. In contrast, under the GDS contract in England, GDPs are required to deliver any and all routine and urgent dental care required *“to keep the mouth, teeth and gums healthy and free of pain”* [33]. These ‘mandatory dental services’ place few, if any *technical* restrictions on the types or volumes of routine treatments that can be offered [33]. Differences in remuneration and public service commitment may also be factors. As of June 2025, DTSS contractors in Ireland are reimbursed to the value of €242 for an oral examination, cleaning and two fillings compared to an average of £84 (€100) for delivering the same (or indeed a higher) volume of care in England [13,34]. Our realist review found that capped (and perceived low) remuneration on public contracts, for example the NHS GDS, incentivised some GDPs to treat large volumes of patients, deliver high volumes of treatments at speed and sometimes ‘cut corners’ to maintain productivity and profitably [15].

On public/private split, a recent NHS England report highlighted that dentists holding GDS contracts dedicated approximately 70% of their clinical time to NHS work [35]. Multivariate analysis also revealed that the more time GDPs spent on NHS work, the lower their levels of motivation were. While no comparative data exists in the Irish context, it is estimated that, inversely, almost two thirds of dental care in Ireland is delivered privately and paid for out of pocket [36]. So, while GDPs reported financial pressures associated with state care delivery in Ireland, these may be less profound than in England and without the same level of health system oversight or pressure to deliver on targets. As highlighted above, the NHS GDS and DTSS contracts differ substantially in their design, baskets of care, remuneration and oversight mechanisms. This limits the direct comparability of GDP experiences across the two systems and therefore interpretation of these cross-system comparisons should be undertaken with caution.

### Strengths and limitations

To the authors’ knowledge, this is the first primary research study to directly address this topic in the Irish context, therefore a notable strength is that it helps fill a significant knowledge gap. By adopting a realist approach to this complex area of inquiry, and utilising CMOCs from the realist review as the initial programme theory, we uncovered a range of high-level system factors acting as modifiable contexts and causal patterns influencing private contractors’ participation in publicly funded dental care in Ireland, providing in-depth analysis specific to that health system. This deepens our understanding of why GDPs are increasingly reluctant to engage in state contracts for the provision of primary oral healthcare to low-income groups. A key strength of this study is that it captures a wide range of perspectives from diverse system actors with expertise and experience at all levels of primary oral healthcare in Ireland including frontline practitioners, health policymakers and health service managers. This allowed for an adequate level of data sufficiency to expand on our initial programme theory and inform our new CMOCs. While the causal patterns identified are based on data which are largely representative of, and therefore specific to Ireland’s oral healthcare system, we have presented our findings at a level of abstraction which is high enough so that they can be applied more widely to other health systems which use contracted care models. It must be noted however, that while interviews generated data which were, from a realist perspective, both contextually and mechanistically rich, they are representative of the opinions, attitudes and experiences of study participants and therefore can be highly subjective, as is common in qualitative methodological approaches [23]. Unlike other realist studies, for example realist evaluations, which may triangulate qualitative findings with quantitative data or documentary analysis, this study was entirely reliant upon qualitative data and the perceived experiences, opinions and attitudes of interviewees, many of which operated within a low-trust context [37]. Caution should therefore be exercised when interpreting these findings as the ultimate truth on this subject. Another limitation of note is that we did not include patients in our recruitment sampling frame owing to this study’s primary focus on the health system and GDPs. While their perspectives are not directly represented in the study’s findings, we did include a patient and public representative with experience accessing the DTSS on our Project Advisory Group.

### Policy implications: ‘Us and them’ – shifting from mutually reinforced disconnection to a trusted shared-learning partnership

Notwithstanding the multitude of health system factors identified, it is the apparent disconnect between GDPs and the health system that appears to permeate through many of the CMOCs and impacts on the functioning of the primary oral healthcare system in Ireland. Specifically, how the dental profession and health system are perceived to engage with one another on various levels, appears to continually reinforce a cycle of mistrust and suspicion which ultimately leads to siloes, impasses, the absence of collaborative working and disconnect between these two key pillars of the health system (leadership/governance and the workforce).

The CMOCs outlined above demonstrate how policy decisions around primary oral healthcare system design and resourcing can result in system fragmentation whereby frontline GDPs are perceived to not be fully embedded within or supported by the health system. As third-party contractors, GDPs are perceived as separate entities, existing at arm’s length to, rather than an integral part of the oral healthcare system in Ireland. These factors, coupled with the perceived deprioritisation of primary oral healthcare during the austerity and COVID-19 pandemic periods, have resulted in GDPs feeling isolated, devalued, not part of, and untrusting of the system. Sustained political and financial commitment to oral health, alongside greater integration of GDPs within public oral healthcare systems, may help counter the reinforcing mechanisms of marginalisation, isolation and mistrust which appear to underpin disengagement from state-funded care.

On system governance and communication, the way in which some GDPs react to health system managers’ attempts at engagement was perceived as being overtly negative and reactionary and is symptomatic of their mistrust and isolation. Conversely, the manner in which health system management engages with GDPs on matters such as prior approvals, probity and patient complaints was frequently perceived as confrontational or dictatorial and only serves to reinforce a culture of mistrust, adversarialism and a lack of symbiosis between the two parties. It was suggested that the language adopted by the health system fuelled negative perceptions, for example the terms *‘probity’* and *‘inspectors’,* and that this was an important context in terms of how GDPs perceive health system actors. Both health systems and GDP interviewees attributed these factors to a culture of adversarialism where the health system is perceived as the *‘police’* and had resulted in further GDP isolation, mistrust, suspicion and unwillingness to engage constructively with the health system. The apparent chasm that exists between the two sides can be summed up in the following quote:

*“it’s definitely an ‘us and them’. They don’t trust us, they don’t think we trust them and we’re not working together”- Interviewee 9*

More transparent and proportionate approaches to governance, communication and quality assurance may help shift perceptions of the health system among GDPs from punitive oversight towards supportive partnership, therefore countering defensiveness, mistrust and professional disengagement.

Away from frontline clinical practice, this disconnect and breakdown in relationships can also be seen at the highest level between health system leadership and professional representatives. Collective negotiations on contracts and system reform were reported to have ended without consensus as a result of what is perceived to be inflexibility and a reluctance to accept concessions or move on set positions. These polarised stances appear to act as reinforcing agents for one another, deepening entrenchment of positions and creating standoffs.

There were perceptions on both sides of a lack of understanding of the challenges faced by each party, for example, running a dental practice as an ongoing concern or implementing a new directive or national policy, and that each side was motivated to a large extent by serving their own interests. The reinstatement of formal consultative and feedback structures, alongside more collaborative approaches to contract negotiation and system reform may help counter polarisation and foster reciprocity, shared ownership and greater trust between the profession and the health system.

GDP non-engagement with state funded dental care is a complex, wicked problem, the aetiology of which lies within a myriad of interwoven health system factors as demonstrated through our CMOCs. Central to this problem is the historical and current tension between the health system and the profession over contractual arrangements to provide publicly funded care (within an increasingly buoyant private market), alongside a requirement for collaborative working in order to reach consensus on service design and ultimately the provision of better care to the population. Resolving this, through for example, clearer contractual arrangements and agreements taking place within formal frameworks or mechanisms that would facilitate transparent, open and collaborative engagement and feedback between the profession and health system (at all levels) could act as a worthwhile starting point for leveraging positive system change. This could potentially unlock opportunities for shared learning, decision making, and partnerships built on mutual understanding and trust where the common goal is increasing timely access to care for patients and reducing oral health inequalities.

## Conclusion

This study identifies a clear causal pathway in which political marginalisation, restrictive governance, and adversarial communication practices shape GDPs’ professional reasoning about their role in public dental systems. Under these conditions, GDPs interpret system structures as constraining to their autonomy, undermining clinical judgement and triggering institutional mistrust. These interpretations, rather than diffuse sentiments, drive disengagement from state-funded oral healthcare systems, particularly within mixed public–private markets where private alternatives are perceived as professionally rewarding.

Crucially, our findings highlight how modifiable system-level levers, namely governance processes, supportive and transparent contract administration, and meaningful consultative structures can potentially reshape how GDPs interpret system intent, mitigating perceived threats to autonomy and rebuilding trust.

## Supporting information

S1 Text Interview Guide. (DOCX)

S2 Text S2 Text and figures. Data excerpts for CMOCs. (DOCX)
