# Supplementary material for: ‘Us and them’: A realist interview study exploring how and why health system factors influence dentists’ participation in state funded, contracted primary dental care for low-income populations in Ireland
Source: PLoS One. 2026 Jul 31;21(7):e0341786. doi: 10.1371/journal.pone.0341786 (PMC13426964; doi:10.1371/journal.pone.0341786)
Supplement: S1 Text — (DOCX) [file pone.0341786.s001.docx]

**S1: Realist Interview Study interview schedule**

So [NAME], once again, thank you very much for agreeing to take part in this interview.

Before we begin, can I just confirm that you have read and understood the PIL, signed the consent form and you agree to take part as per the consent form?

Can I also confirm that you are happy for me to record and transcribe this interview?

Great, so just to clarify, the aim of this study is to understand what are the health system factors that have influenced private GDPs decisions to participate in state-funded, primary oral healthcare schemes in Ireland as contractors to date (or not).

So the way the interview will work is, we’ll begin with some general questions about the oral healthcare system here in Ireland and then we’ll turn to my findings from the international literature review- which I will talk you through as different theories and you will then have the opportunity to comment on, to build upon, refine or refute these, based on your own experience and thinking about the Irish context.

I’m not sure if you’ve had an opportunity to look through the reading material in advance?

If you have any questions, I am happy to answer these now.

**Questions:**

**1.** **Contextualising the role of the interviewee: ask the interviewee about their involvement in / experience or knowledge of and views about state funded contracted primary oral healthcare in Ireland.**

*a. Please describe your current role(s) in primary oral healthcare and what it involves.*

*or*

*Can you tell me what your involvement in / experience of contracted state funded dental care in Ireland has been?*

*b. What are your thoughts/views about state funded dental schemes in Ireland at the present time?*

*c. From your experience, why do you think there has been a reduction in the number of dentists holding DTSS contracts or submitting claims on the DTSS over the past 10 years?*

*d. Conversely, from your experience, why you think the number of GDPs holding PRSI contracts has increased?*

**2. Theory testing and refinement:**

**CMOC 1: Quality care and job satisfaction**

1. Based on your experience and thinking about the Irish system, does this look accurate?
2. Is there any aspect of this that you think isn’t accurate?
3. Is there anything missing?
4. Why do you think this happens?
5. Do you think this is accurate for all GDPs, or does it only happen sometimes or among some GDPs but not others? Why do you think this is?

**Additional prompts:**

Can you give an example from your own experience?

*Some of the research that I have read says that tight/limited state funding often mean restrictions are built into public contracts (to contain costs).*

- *What is your experience of this?*
- *Could you tell me more about that?*
- *Give me an example of where you experienced this or know of this?*

*I have also read that systems or contracts tend to focus on treatment - ‘drilling and filling’ rather than prevention and focus on care delivery by dentists (rather than wider team members)*

- *Is that your experience?*
- *What do you think is the effect of this on how schemes are designed and funded?*

*I’ve read that funding constraints and subsequent contract restrictions can impact on GDP job satisfaction and morale because they feel they can’t deliver high quality, comprehensive care to patients.*

- *What is your experience of that?*
- *Why do you think this happens?*
- *What do you think is a consequence of this among GDPs- can you give an example?*
- *Do you think this/these outcome(s) is/are the same for all GDPs- associates, principals, urban GDPs, rural GDPs, young GDPs, more established GDPs?*

*In what other ways do funding arrangements and contract restrictions affect GDPs’ satisfaction/ ability to practice/offer care to their patients?*

**3. Theory testing and refinement:**

**CMOC 3: Clinical autonomy and control**

1. Based on your experience and thinking about the Irish system, does this look accurate?
2. Is there any aspect of this that you think isn’t accurate?
3. Is there anything missing?
4. Why do you think this happens?
5. Do you think this is accurate for all GDPs, or does it only happen sometimes or among some GDPs but not others? Why do you think this is?

**Additional prompts:**

*Can you give an example from your own experience?*

*Additionally, I have read that for contracts/schemes to be delivered within tight budgets and be standardised, there may be a high degree of health systems oversight or management where GDP local decision making is restricted (for example…).*

- *Is that your experience?*
- *How does this oversight/management affect GDPs?*
- *How do restrictions on clinical decision-making affect GDPs?*
- *What does this lead to? Why do you think this is?*

*I’ve read that contract restrictions can lead to GDPs feeling like they’ve lost clinical control/autonomy or decision-making ability.*

- *What is your experience of that?*
- *Why do you think this happens?*
- *What does this lead to? Why do you think this is?*

**4. Theory testing and refinement:**

**CMOC 4: Business control, consensus, and trust**

1. Based on your experience and thinking about the Irish system, does this look accurate?
2. Is there any aspect of this that you think isn’t accurate?
3. Is there anything missing?
4. Why do you think this happens?
5. Do you think this is accurate for all GDPs, or does it only happen sometimes or among some GDPs but not others? Why do you think this is?

**Additional prompts:**

*Can you give an example from your own experience?*

*What is it about the way [state schemes] or [named state scheme] is/are/was/were changed/administered that made/makes some GDPs feel or think this way?*

*I have also read and been told by others that when changes to contracts (or how they are administered) are made with minimal communication or consensus, this can result in professional opposition, especially when viewed as unfavourable. For example, if fees are reduced or items of service are cut, or prior approval isn’t granted/payments aren’t made.*

- *What is your experience of this?*
- *How does this affect GDPs?*
- *Do you think this changes the way GDPs think or feel about state schemes in any way or those planning or managing these schemes?*
- *Can you provide some examples of this? Is this something you have experience of?*
- *What is it about the way [state schemes] or [named state scheme] is/are/was/were changed/administered that made/makes some GDPs feel or think this way?*

**5. Theory testing and refinement:**

**CMOC 6: The value of State-funded primary dental care**

1. Based on your experience and thinking about the Irish system, does this look accurate?
2. Is there any aspect of this that you think isn’t accurate?
3. Is there anything missing?
4. Why do you think this happens?
5. Do you think this is accurate for all GDPs, or does it only happen sometimes or among some GDPs but not others? Why do you think this is?

**Additional prompts:**

*Can you give an example from your own experience?*
