# Supplementary material for: ‘Us and them’: A realist interview study exploring how and why health system factors influence dentists’ participation in state funded, contracted primary dental care for low-income populations in Ireland
Source: PLoS One. 2026 Jul 31;21(7):e0341786. doi: 10.1371/journal.pone.0341786 (PMC13426964; doi:10.1371/journal.pone.0341786)
Supplement: S2 Text — (DOCX) [file pone.0341786.s002.docx]

**S2: Realist Interview Study illustrative data used to construct CMOCs**


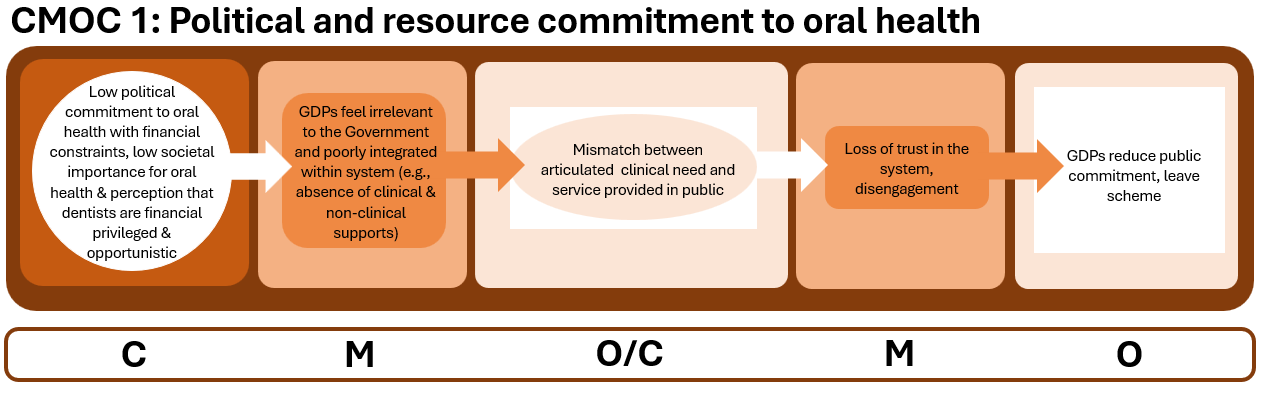


*Dentistry isn't a priority for people. It's very quickly a priority at the weekend if they're in trouble. And I think, I was really shocked during the recession when the PRSI scheme was cut right back, and the public didn't object. I thought that was, that was a great failure, actually, and I think, I think our public let us down. Now, why? I don't know, or maybe, maybe there, maybe there needed to be an unseen hand there that that encouraged them to to object. Maybe we should have worked through unions or something, but we didn't. But that was, that was shocking – Interviewee 14*

*And in the end of the day, there's almost no political capital from an improved primary prevention system, and there's huge political capital cutting the tape on a new hospital facility. And you know that I think we are still a long way from having an understanding as a population of what a good health service would look like, because it's so focused on the visible crises in the dysfunction of the current arrangements, as opposed to being a pathway to improved functioning across all levels in the system – Interviewee 2*

*Dentistry is of no importance whatsoever to government and…… I think it's it's proven in the fact, of the funding for it like you know what I mean that there is………it's been allowed to kind of stagnate, just wither on the vine like you know – Interviewee 17*

*I think the budget in 2010 - they took kind of a guillotine to the to the public dental schemes, both of them, overnight. And I think a lot of the professionals would have compared themselves to other, you know, maybe, if you look at medical GPs, because pharmacies, you know, those cuts, dental was, you know, disproportionately impacted so they were, you know, ditched by the government effectively in in 2010 so back then we have the bad, recessionary times and private practices, I personally, you know, really, really struggling with, with an overnight reduction of 70% of their budgets – Interviewee 5*

*And then we have the COVID 19. And we have the, for instance, you know, in Scotland, they paid NHS dentists their supports through COVID and for a significant time after, just to make sure that they retained interest in terms of practicing NHS dentistry. And here, not only were there an absence of supports, but this the PPE element. It sounds like a small thing, but in the grand scheme of things, it was a mark of disrespect, I think that was felt by a lot of dentists, and in terms of their isolation. And then this whole question of, you know, closing my practice, what am I going to do? Is this essential dentistry? What's essential dentistry? When am I opening? No guidance, no support from either of the institutions in the health system and then, you know, you re-enter ‘normal life’ again, and you have no update on fee schedule, really – Interviewee 5*

*You are a country, and I suggest you go to the World Bank data, which is per capita in terms of GDP, one of the best paid in Europe and your government has consistently refused to fund decent or healthcare, and that, to me, is an utter disgrace – Interviewee 11*

*I think there was, you know, there was a promise of, this was actually a thing that got people very annoyed - there was a promise of PPE during COVID. You had to, you know, declare that you were a medical card practitioner and you were promised PPE. That never materialized. And I think that was the last straw for a lot of people, but it was certainly a big problem for me. I mean, I could, I had my own PPE, and I could get my own PPE, and I was planning for it, and I was okay, but the fact that, you know, we weren't relevant in the whole scheme of things, was quite difficult, and I think that was a turning point for a lot of people – Interviewee 19*

*But I think a lot of them have left the scheme because there's no there's also a lack of support or acknowledgement from us in the HSE, we no longer have local monitoring committees – Interviewee 3*

*And so, yeah, I mean, there's so many issues, and then lack of places to refer, you know, like there's very ad hoc arrangements and for referrals, and it's different. What we have here is different to what I had in [REGION]. And in some areas, there's nowhere to refer patients. So if you have an oral surgery case or a complex root canal treatment or, you know, there's nowhere to go, and there's no funding for that. So I think the whole, the whole system is just, is just inadequate – Interviewee 9*


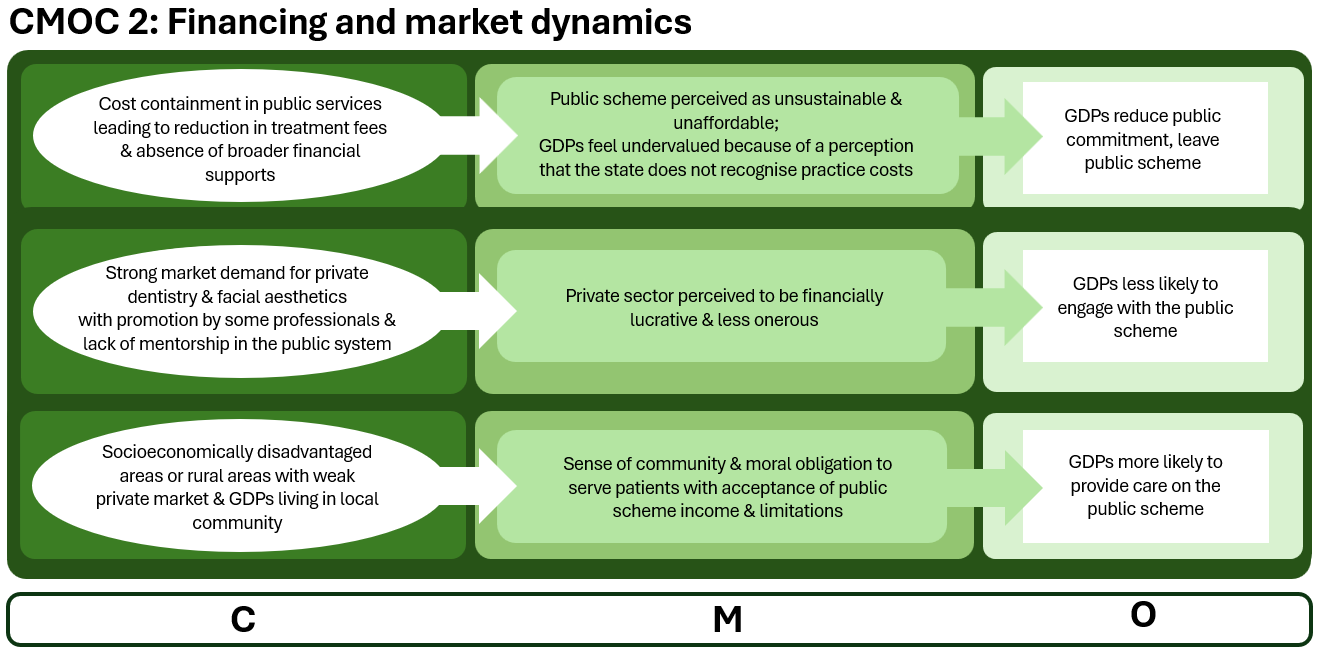


*Yeah, I mean the fees, it's getting to the lower end, you know, of…. I mean, when it was 50 euros for a composite, just a small few years ago, I was just, you know, I was feeling very, you know, a lot of antipathy towards the scheme. But when up to 80 all was forgiven, you know…but its starting to feel a bit painful again – Interviewee 16*

*When it starts costing them money to provide this service, then it becomes unsustainable and that's what happened with regards to some dentists that left the scheme. But if they are still kind of making, and at the end of the day the dentistry's a business as well as being a healthcare profession, if it doesn't make business sense for dentists to do something (laughs) you know you're going to have difficulty to get them to do it, you know, so it has to make business sense – Interviewee 1*

*I'd say the third thing that's reduced it is that there's easier money elsewhere with Botox, and actually quite a lot of you know, a lot of practices that I visit now are doing Botox as well. So they're spending their time, essentially wasting their time doing this thing. But it's lucrative. It's not dentistry – Interviewee 18*

*People are interested in the ‘white/shiny’…… they would pay money for the ‘white/shiny’ so, and it's not that there's anything wrong with actually having a nice white smile or having nice straight teeth, but it's easier money than dealing with the provision of treatment for gum disease, treatment for dental caries, and trying to improve people's actual physical oral health– Interviewee 10*

*The context still plays into this, I think. So you'll do it, and you'll suck it up if you have no other patients coming in the door. So, you know, we would have happily seen medical card patients and been delighted to have the guaranteed money come into the bank when the economy was poor and when there wasn't a lot of private practice business, and when the wheel starts to shift to when you have another option, then, you know, it becomes far more appetizing to say, okay, look, why am I bothering?– Interviewee 5*

*Ah yeah, I mean, I would have left the scheme if I could, but I can't, because of my location, you know (laughs). But, you know, I think ..what I'm finding later in life is…. you know, the most satisfying part of the job is giving everybody access, you know what I mean. Its….now look,….I can't leave the scheme, so I'm not Mother Theresa but I’m kinda, I'm trying to……reflect….on…..you know….and it's the hard cases..they’re coming down, they cant… I mean, it would be tragic if these people didn't have access locally…. to the scheme. And… you know…. it is…. it is satisfying. More satisfying than if you just did your private work…its just going, is just going wow, can we operate the scheme… that worked very smoothly for that person…that’s another happy customer. And also, I think maybe in universities, people should, you know, students and someone came in and said, look the state has…..subsidised a huge sum of your training to become a dentist …to the tune of 100s of 1000s. So….. you do have an obligation to the state as well to be sympathetic to those on the schemes. That’s something I get now…the state did subsidise my education a huge amount. So we have a mutual obligation to the state…its just another little thing I tell myself of myself to get me through the day – Interviewee 16*

*I think if I answered it initially, the opposite, why are why is anyone staying? I think the majority of people who are staying are staying out of a moral obligation, because they feel, particularly if they're in small towns, they feel like they've treated these people for years. They just can't go off the medical card. There's nobody else who are going to be who's going to take these patients, you know, and like, I know, a few practices, and there are dentists who are well off financially and are years working, and they say they financially, they're not making money on the medical card holders, but they just would feel too bad to leave it. So they're staying for moral reasons - some people – Interviewee 9*

**
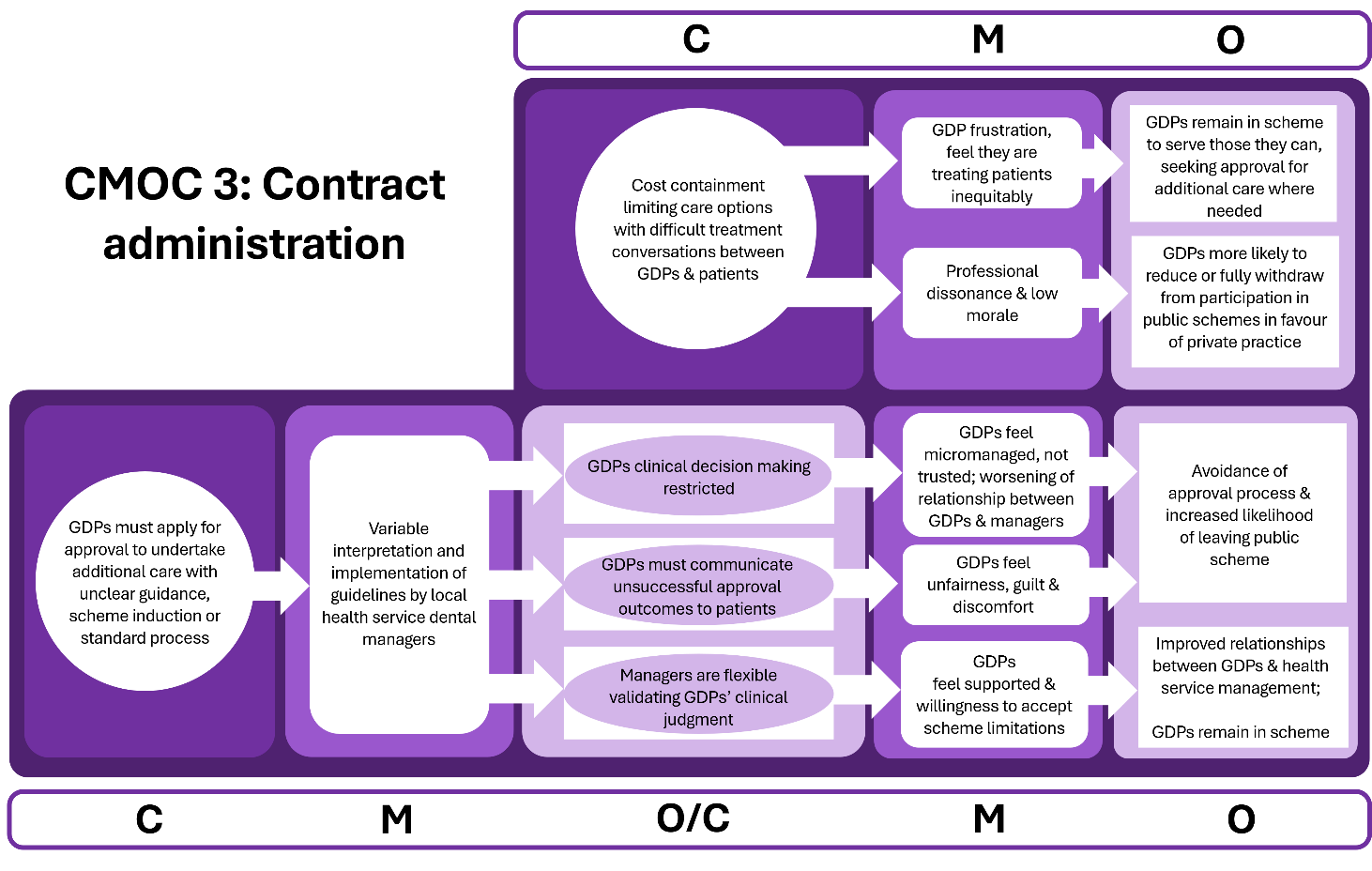
**

*I certainly went into dentistry to try and A) do the right thing, and B) help people. And if, if you're hampered in your ability to do that, that's very difficult…. it's extremely challenging not to be able to give the patients the care that A) you want and B) they need. Very challenging. You know, particularly when patients can't afford it. That’s one of the reasons I've continued with the medical card scheme – Interviewee 19*

*I think that being in a situation when, when, when it was reduced to two fillings per year like it just, you know it it really it really it was terrible really. It it it just, you know you were leaving active disease like and you were telling patients there's active disease here. There's active caries, there's active gum disease and then you know a lot of these patients may have lost some teeth and then perversely then they were looking for a partial denture or they were looking for you know, and and none of the research supports putting a partial denture into a mouth with active disease. And you know, you just, it was just soul destroying – Interviewee 17*

*Well, it wears you down, and it takes them a lot of time, you know. So it's, it's time that could be spent discussing patients issues or problems, or, you know, potential treatment options. Instead of discussing all that positive stuff or preventive regimes, instead of doing that, we're explaining a system that is not fit for purpose, you know. And that's not really what we're there for, you know? – Interviewee 8*

*Information is a big problem here, because they get the medical card and they don't know what they're entitled to. They go into the dentist, and the dentist says you'll only get that if the HSE approves it, and you won't get that because it's not available on the scheme, and they don't know where that's written down. They don't have any information. They go looking online, they can't find it, and then they get annoyed. And then the HSE may decide, the local principal, may decide, I'm not going to approve it. Then they're going, ‘well, I want, I can't afford, I'm not going to pay for this’. Then they want to appeal it. There's no appeals mechanism. Where does that go, you know. And so the poor old clinician is left with it is the person who's dealing with the irate patient – Interviewee 3*

*Another thing is sending for approval. That's annoying. You know, that's that's pretty annoying. It's like, why do I have to send for approval? If a patient needs dentures, they need the dentures. If their dentures are poor fitting, they're rocking in their mouth, they should get a new set of dentures, regardless of them being made in the last five years or not. So I definitely think the sending for approval is annoying. It's not the worst, but it does get the way it makes you feel like you're not in control of the treatment. Someone always up there, ‘micromanaging’ everything. It's annoying – Interviewee 13*

*Yeah, again, it kind of reminds me of when the principal dentist was suggesting a filling didn’t need doing…. very annoying, but it was only briefly there. But yes, I would find it very annoying if they said, they were saying, you know, why don’t you go for a full denture here instead of a partial so….but luckily, they don't seem to do that, you know. But that would be, if.. that would be my take on that would be, that would be a ‘no, no’, you know, if they were being a bit too…. ‘oversight-y’ on clinical decisions. How do they do that remotely? Do you know what I mean….very annoying – Interviewee 16*

*I think probably the rigidity of it and the paperwork involved is probably something as well. It was the reason I never really got into it, because my local principal was a thorny sort of a person, and the applying for approval for certain treatments, I was fairly sure would have run into problems. So, yeah, the red tape, I think, as well, dentists tend to be quite strong, strong headed people, in my experience, and they don't like being told what they can and can't do – Interviewee 8*

*Someone ‘up there’ is saying, ‘No’… they're not approving, you know, and like, it leaves it in your hands to deliver that news to the patient. It's unfair, because you know the patient needs the treatment and they're not getting it, and it's unfair on the dentist to be like, ‘Oh I'm so sorry you weren't approved for the treatment. You have to pay X amount of money to get it on the private scheme’. You know, it's really annoying. So that definitely is… it can lead to the GDP feeling lost, a lack of clinical autonomy, it, it makes you feel like I'm being ‘micromanaged’, and it's, like I'm self employed, you know, I'm a dentist, and I’m self employed. I shouldn't be feeling like I'm being micromanaged. But it does make you feel like you are being micromanaged, like every decision you're making is being honed in, does the does the person, does the patient deserve this, or are they eligible for this or not? That's not fair ­– Interviewee 13*

*I am inclined to ensure that within what they are eligible to provide, that there is as little restriction or limitation or hindrance on our behalf put on those dentists as possible. Because, you know, we are trying to keep the last man, you know, the last man standing, in effect. But you know, the HSE aren't stupid, the principals know that we’re at the mercy of the dentists. And so I would be very surprised if, if you looked nationally, although there may be variability, I think there will certainly be a lot of, kind of, really serious efforts made to try and keep this moving as quickly as possible, because otherwise it falls back on the HSE. And I think ultimately that maybe is what needs to happen. But that's maybe a different discussion – Interviewee 5*

*No, no guidance is given. Do you know what I mean? There's no guidance given to you. They send you a booklet and then a few sheets in your application form back, but there's no real, actual guidance when you're reading the booklet, you know exactly what is going to be approved or not. I feel like they keep that very hidden. I'm not sure, but I do feel like they keep it ‘under wraps’, and they approve whatever they think should be approved, which is not really nice. But I heard that from my nurses, actually, who have been in the practice for long, working with other dentists. And they would tell me, put down any systemic disease, put down that they have diabetes or depression or, I don't know, you know. But in the first few, three to four months, it was in my head, I was like, two fillings only, and I'm really sorry, etc, but I didn't realize they would actually approve some” – Interviewee 13*

**
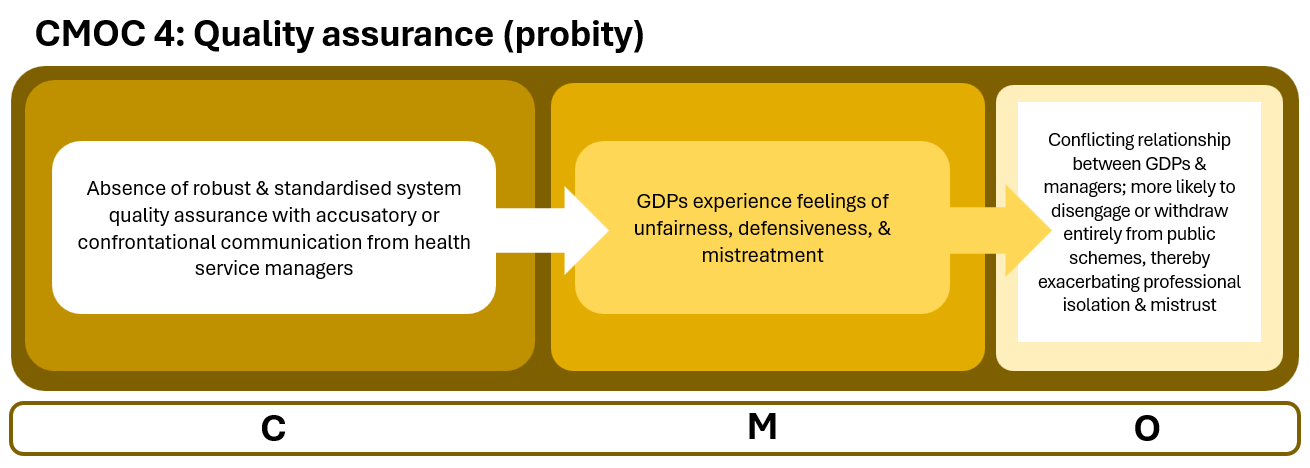
**

*And I think that broad, sweeping letters, sometimes with confrontational statements in them. Sometimes, I have heard a couple of people to be more kind of, I suppose, dental colleagues who I know from kind of college years or whatever, saying ‘that letter now has finished me, I'm leaving’ – Interviewee 9*

*I think people are, and if I didn't say it earlier, I think people are more inclined actually, to leave the medical card scheme because they are fed up with the HSE…. than with the dentistry. They also, you know, people have said they were fed up they weren't getting paid on time. People have said that they were fed up with, like, you know, these kind of probity questions coming at dentists who were good dentists, who were not messing the system, you know – Interviewee 9*

*there was an examining dentist scheme that, you know was starting….. I know most of the dentists involved in the examining dentist scheme when they were very frustrated like that they could see that that, this would be fruitful and they were trained, etcetera and this was a, this was a big hope for the profession as well, like, but that fell away as well, like you know, so – Interviewee 17*

*Now, there was a time when, I think the contractors, the dentists that were providing the DTSS, there was a time when there were a little bit more consultation was going on, there was a little bit more feedback to them. But now I think the only feedback they get is maybe a letter from us in the inspectorate asking them to explain, you know, certain things under claim, claiming patterns, or a phone call asking them to explain or to answer to a complaint, you know – Interviewee 3*

*But I think if you had a more low level advisory kind of , the HSE had a more, came in at that little bit, worked with the clinicians, you know, and there was a little bit more, I don't like ‘peer review’, but a little bit more kind of informal- formal kind of relationship. The scheme would work better – Interviewee 3*

*People have said that they were fed up with, like, you know, these kind of probity questions coming at dentists who were good dentists, who were not messing the system, you know. And so, you know, I feel, yeah, despondency, with the HSE, feeling that they weren't heard, ‘Nobody's listening to us’, ‘We're not supported’, ‘I'm fed up with this’, ‘Now I'm going’ – Interviewee 9*

*It was never set up like that. You know, there were, this was contracted out, and it was always a ‘them and us’ sort of scenario. And you know, the HSE, it's an adversarial relationship in a lot of cases. Yes, you know, that's kind of watching what people are claiming – Interviewee 9*

*But when you're looking at the finance data in the DTSS, there was absolutely no doubt whatsoever that some of the trends of surgical versus non-surgical extractions were pointing towards over claiming for surgical extractions, you know, 100% surgical extractions versus zero. Now, how that was managed, and the language around it, and the interactions with the people, the ‘people side’ of dealing with that probity……may not have been as positively managed, for want of a better description, as it might have been – Interviewee 10*


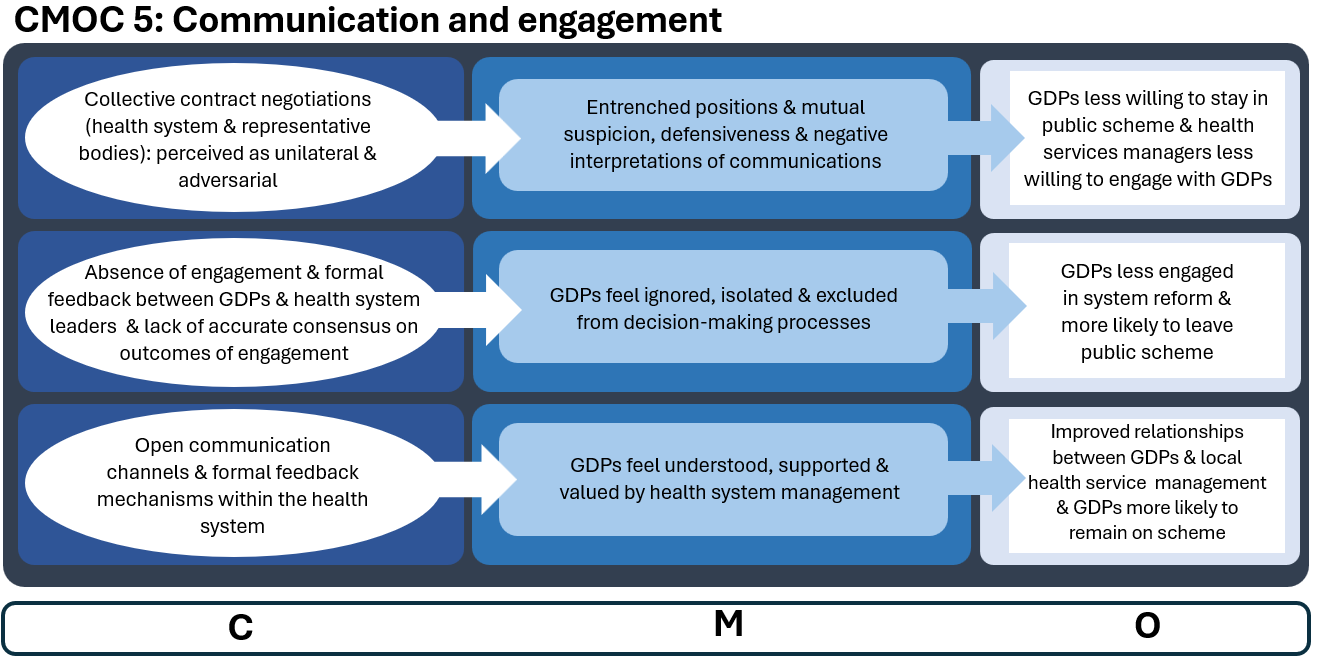


*And I've heard, I've heard it from people who were in negotiations with, I think it was the HSE at the time, and the conversation that was alleged was that at the start of the meeting, the HSE people said, ‘right, well, we can this is what's on the table, and we can talk about it for a week, but nothing in is going to go on the table other than what we're putting on the table now. So you can take it or leave it, or we can sit here for a week and talk about what you want to do’ so that, like, that's that came from somebody who was there. They didn't have a particular reason for telling me that story, that they were there. That's what happened. So if that's, if that's the level, you can see where mistrust, you know. So the mistrust from the health HSE side came from what I would perceive to be there, their perception anyway, of over claiming and abuse of the system, and then the mistrust on the dental side. What was well, like this just, we're just getting told that this is happening and there's nothing we can do about it. So, yeah, communication – Interviewee 8*

*I can understand dentist getting demotivated. Demotivated because my experience of the means of communication, of changes in this game, it's not always great. Hasn't been great. It's been kind of, ‘well, here you go’, ‘This is what's happening, if you think you can do anything about good luck to you’ – Interviewee 8*

*I suppose…I think there needs to be, how would I put this, there needs to be more cross contamination between the public sector and the private sector There needs to be a lot more communication on both sides. This kind of everyone sticking to their own side of the fence is not helpful for anybody. That's my own feeling. Just whether it's a lack of trust or a lack of communication or a lack of openness on either or both sides that part, I can't really figure out, but I do know there's a big divide, and there is a lack of understanding on both sides of the fence – Interviewee 19*

*And I mean, I think, I think the clinicians, they've been so much pushed out of any kind of consultative process in my mind, that I kind of understand why some of them have left the scheme, you know – Interviewee 3*

*And then I think maybe a little bit of ownership over a system change, you know, like they are so isolated in terms of development of policy in terms of, you know, the PPE crisis and controversy during COVID, and I think that was, you know, again, a key kind of turning point in terms of their engagement with the system. I think a lot of the, you the best place to advise, but this idea of a shared ownership over a system will give you some kind of pride in the system and kind of a responsibility to it – Interviewee 5*

*And I think there must be a responsibility on dentists also to be willing to engage and to be willing to at least have a discussion with…. if the opportunity is offered to them. I think the issue is that the opportunity maybe has not been offered to them, or maybe, I don't know, I'm not privy to that information, but I do think you know, there can be an attitude of, ‘I want everything, but I'm not going to, you know, really engage at any meaningful level either’. You know, change is difficult for everybody. We can't have everything in a publicly funded scheme. It's not going to work like that. We should be considered, and we should be part of the of the process…. so I think it's a really…. at the minute, I think it's quite a fractured environment – Interviewee 5*

*I actually found the relationship, I could pick up the phone and have the chat, which was a big help - having a conversation, a direct conversation by phone helps enormously for everyone to know exactly where everybody else is up to. Yeah, I would be a great believer in picking up the phone and having a chat – Interviewee 19*
