# Supplementary material for: ‘Us and them’: A realist interview study exploring how and why health system factors influence dentists’ participation in state funded, contracted primary dental care for low-income populations in Ireland
Source: PLoS One. 2026 Jul 31;21(7):e0341786. doi: 10.1371/journal.pone.0341786 (PMC13426964; doi:10.1371/journal.pone.0341786)
Supplement: Attachment — Submitted filename: Response to Reviewers.docx [file pone.0341786.s004.docx]

| **Reviewer #1** | |
| --- | --- |
| **Comment/query** | **Response** |
| **Why only six frontline GDPs were included?**  **Whether this number was determined by theoretical sufficiency.**  **How “theoretical sufficiency” was operationalised in the context of realist interviews.** | *Thank you for your question and observations. As this was a theory driven realist study, we followed the RAMESES II guidance on realist interviews. In realist research, sampling is theory-driven rather than determined by numerical thresholds. Realist interviews aim to test and refine emerging programme theory by engaging participants who can provide relevant insights into contexts and mechanisms under study. Accordingly, a sampling framework was used to recruit frontline GDPs with varying levels of experience working under the DTSS contract in Ireland (as well as years’ experience, urban/rural practice). This ensured inclusion of practitioners with different levels of exposure to and engagement with that contract.*  *While we were able to recruit 6 frontline GDPs, with additional time and resource capacity, we would have liked to recruit more participants however we found it challenging engaging/recruiting frontline GDPs for the study. From a practical and pragmatic perspective, we felt that there was enough within the 6 GDP (and other) interviews to give us sufficiency of data to develop realist programme theory that addressed our research aim and which might inform policy and practice decision-making, rather than to create complete theory in this regard. The theory we have sought to produce is not intended to represent the definitive theory or final ‘truth’ on this subject matter but rather it proposes theory that can be refined and expanded on through future research.*  *Regarding theoretical sufficiency, recruitment continued until interviews no longer contributed substantively new insights to the refinement of the emerging programme theory. The six GDP interviews generated sufficiently rich accounts across practitioners with diverse contractual experience to support interrogation and refinement of programme theory, consistent with realist interviewing principles. We acknowledge that recruiting more GDPs may have provided new insights but we feel were able to collect enough data to inform the development of programme theory based on these 6 interviews (and others).* |
| **It would be helpful if the authors could elaborate on:  How they determined that sufficient variation had been achieved,** | *This study captured a wide range of perspectives from diverse system actors with expertise and experience at all levels of primary oral healthcare in Ireland including frontline GDPs and health service leaders and managers. This allowed for an adequate level of data variation to expand on our initial programme theory and inform our new CMOCs. We did not have a quantitative way of measuring variation therefore we looked for illustrative cases/data to inform the development of realist theory.*  *According to Dey (1999), the point at which the researcher has gathered enough data of ‘conceptual depth or density’ to inform the development of emerging theory and where the gathering of any additional data will not enlighten any new insights is referred to as ‘theoretical sufficiency’. We believe that, based on the demi-regularities within the interview data, that is, the consistency with which interviewees were roundly discussing the same issues, that there were no new insights, contexts or mechanisms to be gleaned from additional interviews and that we had captured enough data to inform policy-relevant programme theory.*  *Other interview-based realist studies interviewed similar number of participants across carious sectors, for example, e.g., Siersbaek et al., 2023 (*[*https://doi.org/10.1186/s12939-023-02029-8*](https://doi.org/10.1186/s12939-023-02029-8)*) interviewed 12 people, Jagosh et al., 2015 (*[*https://doi.org/10.1186/s12889-015-1949-1*](https://doi.org/10.1186/s12889-015-1949-1)*) interviewed 24 people.*  *According to RAMESES, sampling for realist interviews is theory based. That is, respondents are selected because they are in a position to cast light on a hypothesis or a particular aspect of programme theory. Realist hypotheses are not confirmed or abandoned through “saturation” in interviews, but using multiple types of data obtained in a multi-method strategy. At the design stages of realist evaluation projects, it can be difficult to establish a definite number of interviews required.* |
| ***Whether new mechanisms ceased to emerge, and how sufficiency was judged within the iterative realist interview process.*** | *We analysed the data until no substantive new insights were forthcoming. We acknowledge that had we recruited and interviewed more people, there would no doubt have been more data however, from the interviews that had been conducted, we believe we had captured enough data to develop realist programme theory that was both policy and practice-relevant. We acknowledge that were practical and pragmatic reasons underpinning our decision to interview 6 GDPs, which, we ultimately balanced against our ability to inform programme theory that was policy and practice-relevant and therefore would address our research aim.* |
| **Given the theory-driven nature of realist interviews, reflexivity is particularly important. The manuscript would benefit from clarification regarding:**  ***How the interviewer’s prior knowledge and involvement in the earlier realist review may have influenced the interview process.***  ***Whether reflexive journaling or other strategies were used to monitor interpretive influence.*** | *Thank you for this very important observation and we are in complete agreement that prior experiences and knowledge can play an important role in influencing the process.*  *Realist interviews are explicitly theory-driven and the interviewer’s knowledge of the emerging programme theory is an expected and necessary component of the process. As described in the RAMESES II guidance, realist interviews operationalise through the “teacher–learner cycle,” in which the interviewer introduces elements of the developing programme theory and invites participants to confirm, challenge, or refine these ideas based on their experience. In this approach, the interviewer puts forward provisional explanations to stimulate conversation and test emerging theory.*  *Accordingly, the interviewer’s prior involvement in the earlier realist review informed the development of CMOCs used (initial programme theory) and interview prompts, which were used to structure discussion and elicit interviewees’ perspectives on whether these explanations resonated with their experience. Rather than representing a source of bias to be removed, this prior knowledge is consistent with realist interviewing, where emerging interpretations are intentionally shared with participants and subjected to scrutiny.*  *Reflexivity was therefore embedded within the teacher–learner dialogue itself, as participants were invited to critique, nuance, or reject the proposed explanations. This process enabled continual testing of the interviewer’s interpretations against participants’ experiential accounts, ensuring that programme theory refinement remained grounded in practitioners’ perspectives, consistent with the RAMESES II realist interviewing guidance.*  *In order to mitigate against biases, several strategies were adopted. Firstly, senior supervising co-authors were non-dentists with experience in conducting realist studies. Throughout the interview process, regular meetings were held with senior supervising co-authors to discuss emerging findings, check biases and encourage reflexive practice.*  *Additionally, from the outset, the RAMESES II realist interviewing guidelines by Manzano were utilised as the basis for developing interview schedules. These schedules commenced with broad, open questions to elucidate new CMOCs different from the 4 used from the realist review. Thereafter, questioning followed the same broad structure, asking interviewees to what extent they agree/disagreed/could relate to CMOCs and give examples of the each of the 4 CMOCs. Questions were based on RAMESES realist interview ‘starter set’ of questions so were structured around these. Please see interview schedule in revised supplemental. Prior to commencing interviews, draft interview schedules were reviewed by senior supervising co-authors to ensure suitability, followed by piloting among 3 non-dental researchers.*  *Regarding reflexivity specifically, we have added the following text to aid readers:*  ***A reflexive diary was maintained by the PI after interviews. This facilitated reflective awareness of potential internal and external influences on data interpretation, including professional roles and the ethical implications of being a clinician-researcher, as these could shape how the data were interpreted. To support objectivity and minimise bias, the data were continually reviewed and independently checked by senior supervising co-authors throughout the analysis and synthesis.*** |
| **Further methodological transparency would strengthen the realist contribution. Specifically:**  ***Were CMOCs refined iteratively across interviews?***  ***Were conflicting or competing CMOCs retained and explored?***  ***How were the 13 individual CMOCs consolidated into five higher-level CMOCs?***  ***Providing a brief explanation of this consolidation process would enhance analytic clarity.*** | *Many thanks for your questions on methodological transparency. We can confirm that the original four CMOCs from the realist review were explored as part of this study however these were used as a ‘springboard’/conversation starter for realist interviews. CMOCs arising from a review are rarely expected to be replicated in an interview study. They are provisional programme theories from the international literature. Using the four review CMOCs as starting points to explore how contexts and mechanisms operated within the Irish system is consistent with realist logic.*  *To aid readers, we have added the following text to the newly named* ***‘Initial Programme Theory’*** *section of the methods:*  ***Consistent with realist logic, the four CMOCs from the realist review represented provisional theories from the international literature which were used as a ‘springboard’ from which to explore how contexts and mechanisms operate within the Irish system. This interview study allowed us to adapt and expand these CMOCs through a process of theory testing and refinement, rather than just theory confirmation.***  *Our realist interview CMOCs therefore look different to our review CMOCs. The fact that our interview CMOCs look different does not necessarily mean they are unrelated or in conflict. It reflects the process of theory refinement. Realist work is iterative with theories being tested, refined, reconfigured, and sometimes repackaged as understanding deepens. This formed part of the theory refinement process, rather than theory confirmation. We selected the four CMOCs as the most transferable and plausible starting points, and the interview study allowed us to specify, adapt and contextualise them. Additionally, the data used to generate the CMOCs are very different – international published literature versus Irish interviews and therefore it is not unexpected that they are different.*  *The 13 individual CMOCs are presented in abstracted format. This was deemed appropriate in the context of allowing these to be clearly understood and indeed tested elsewhere by international audiences. These were subsequently consolidated into the 5 CMOCs but presented specifically in action in the Irish context. The content within the 5 consolidated CMOC is that of the 13 individual CMOCs but presented differently and based around the broad themes or concepts under which these were felt to fall under by the research team.*  *The entire process of building, iteratively refining and ultimately crafting the 13 and then consolidating into 5 consolidated CMOCs took place through early and often sharing of contexts/mechanisms/outcomes between the PI and senior supervising authors (using a series of online shared documents (Microsoft word and PowerPoint for Visualisation), regular meetings to discuss and refine CMOCs) and finally our PAG workshop with external stakeholders.*  *To aid readers, the following paragraph has been added to the newly renamed* ***‘Coding, analysis and synthesis’*** *section:*  ***The process of building, iteratively refining and crafting CMOCs took place through early and often sharing of emerging contexts, mechanisms and outcomes using a series of online shared documents (Microsoft Word and PowerPoint for data visualisation), and through regular meetings where CMOCs were discussed and refined. These processes, built in to the research served as a means of quality assurance which were not quantitative, but rather iterative and dynamic in nature. This process resulted in 13 individual CMOCs presented in abstracted format. Thereafter, these were consolidated into 5 CMOCs specific to the Irish context and based around the broad themes or concepts under which these were felt to fall under by the research team.*** |
| **5. Results** – Differentiation of Context, Mechanism, and Outcome  The Results are rich and policy-relevant, and the five consolidated CMOCs form a coherent explanatory account.  ***To further strengthen the realist contribution, the manuscript would benefit from clearer and more consistent differentiation between:  Contexts, Mechanisms (reasoning/resources), and Outcomes.***  ***Several constructs (e.g., trust, demotivation, fragmentation) appear to function in multiple roles across CMOCs. Clarifying how these were categorised would improve conceptual precision.*** | Many thanks for you comments and suggestions for strengthening this paper further. To assist readers, we have added the following explanatory paragraph in the methods section to more clearly articulate the meaning of context, mechanism and outcome in scientific realism:  ***In realist research, outcomes are products of interactions between contexts (C) and mechanisms (O). ‘Context’ refers not only to physical settings, but a broader set of conditions in which a phenomenon occurs, including individual characteristics, interpersonal relationships, institutional norms, and wider political, socio-economic and cultural settings or circumstances. Contexts shape whether and how programmes work by influencing the activation of mechanisms. ‘Mechanisms’ (M) are underlying, often hidden generative processes, such as individuals’ reasoning and responses which are triggered in certain contexts, resulting in outcomes of interest. ‘Outcomes’ are the intended and unintended consequences that arise when mechanisms are activated in specific contexts; these may be proximal or distal and tend to follow semi-regular patterns, though variation is expected given the complexity of social systems.***  *Regarding the differentiation between reasoning/resources, as this was a ‘diagnostic’ realist interview study focussed on understanding a health systems problem (rather than a traditional realist evaluation seeking to evaluate a new or specified intervention or policy), the resources are less apparent therefore the mechanisms lean more towards reasoning in this study. Specifically, these tend to relate to how GDPs respond to conditions - not just their feelings, but also the complex dynamic between the State/policymakers/professional unions as reported by interviewees, and the dynamic between stakeholders. We have, however, reviewed our CMOCs and made amendments to these where possible to provide further clarify in the wording around these mechanisms.*  *Regarding several constructs appearing across CMOCs, to provide additional clarity to readers, we have reviewed all CMOCs and where appropriate, added additional text to clarify, differentiate or make more explicit how these different constructs function within our CMOCs, for example (additional text in green):*  *“The way in which health system management communicates with GDPs on probity (and other issues such as patient complaints) and the language adopted was perceived by GDPs (and indeed some within HSE management) as unfriendly, confrontational and accusatory, which contributed to feelings of wrongdoing among dentists, mistrust* ***in*** *and* ***suspicion of the health system among GDPs****.”* |
| ***Additionally, some potentially contentious claims (e.g., references to cultural opportunism or perceptions of accusatory tone) would be strengthened by clarifying:***  ***Whether these views were widely shared or contested across stakeholder groups, and  How disconfirming or divergent cases informed programme theory refinement.*** | *Yes, claims made with regards to cultural opportunism and perceptions of accusatory tone were reported in a pattern of regularity across interviews. These demi-regularities within the data are in keeping with realist research wherein patterns within the data are observed and inform theory development and refinement, while recognising there will always be some divergent cases.*  *As part of our data analysis and synthesis of CMOCs and programme theory, we compared and contrasted divergent data emerging from interviews, identifying demiregularites and middle range theories. We acknowledge, that with a larger sample size, there may have been more divergent cases. Additionally, as this was a realist study, it does not represent the final word or definitive ‘truth’ on our chosen topic, rather we have produced theory which can be further explored and built on going forward in other research.*  *To highlight the examples raised in your point, below are some illustrative quotes highlighting the patterns of regularity with which interviewees reported on respective contentious issues:*  Cultural opportunism:  *But when you're looking at the finance data in the DTSS, there was absolutely no doubt whatsoever that some of the trends of surgical versus non-surgical extractions were pointing towards over claiming for surgical extractions, you know, 100% surgical extractions versus zero. Now, how that was managed, and the language around it, and the interactions with the people, the ‘people side’ of dealing with that probity……may not have been as positively managed, for want of a better description, as it might have been.*  *Yeah well, my first thought on the system here always was that we were under examined or under checked, yeah. I think having worked in in England, the DPB called patients in for regular checks, yeah, to see was the work. So the whole reason my kind of, the biggest indicator for me that this worked in England, was how fissure sealants were made white so they could be checked by the DPB. You know, they weren't tooth coloured anymore, so at one stage they were so they would call my patients in regularly. I probably get a letter with five names on it once a month to say we are calling these people. So I knew at all points that I was being checked, so that kept me in in order. When I worked there, I never felt we had that property here, I know towards the end of the decent fee for item, or half decent fee per item, it started to come in. But I think a lot of dentists, or I know haven't spoken to some dentists, they did abuse the scheme. They fucking… you know, it annoyed me, because they claimed for stuff they didn't do. They overclaimed for stuff that they did do. Sort of ruined it. So I think had we had a proper oversight, it might have kept people in check, but that never came in, and the oversight that came in was front loaded. So it almost felt to me like there was an assumption of fraud before you actually did the treatment, rather than the proper fraudulent people being taken down. Now, some people did get taken down. I know that some people got put on prior approval, but it seemed to happen very late in the scheme. There seemed to be a kind of a naivety to in the early 2000s that, like, people were allowed to ‘run riot’ for quite a while.*  *So I wish I would have liked that there was like, it always astounds me how, and you can see it looking at the literature, there are models in existence for this stuff going back decades. Yeah, we seem to go into some of these things, particularly in Ireland, with a naivety that's hard to understand. Yeah, look at the international models, look at the mistakes they made, copy, the good things, leave out the bad things, you know? So, yeah, the external oversight and accountability, I think, was very lacking. And then, as I said, there was a sort of this assumption that we were all over to it, so we're going to hit you early, and we're gonna restrict what you are allowed to do, because you're gonna scam the system anyway. That's the way I felt.*  *I think it could be a little bit of ‘small country syndrome’ where we don't really, we don't like, fundamentally, all Irish people have a sort of a default for a bit of ‘jiggery pokery’ and a little bit of ‘duck and a dive’, like we just had, kind of have that. I think it's made it might be post colonial, I don't know, but we definitely have a system. We have a tendency to try to get around systems. So my opinion of that is, if we're setting up a system in our country, it has to be water tight, because there will be a certain percentage of people who will abuse it. So I think that you know, in hindsight, or even for the future, I think a proper system of checks and balances should be brought in to make the system fair for everybody. Because we need, like, Irish people, I think, need structures, and we need regulation, yeah, for systems to work well.*  *So it was it was right and there was another strand to all this, and I suppose I am being recorded and it is confidential, but it was the ‘good guys’ and there was the ‘bad guys’ and the good guys were the guys that did the treatment that was necessary; took the X-rays, did the diagnosis, did it to a reasonable standard functional, and there was the ‘bad guys’ and the bad guys were, the what we anecdotally would be called the ‘schemers’ and the schemers were the guys that a patient would would come back to me and say I've been told I need X done and Y&Z and then I took my bite-wings and I do a diagnosis and I say well, I can see X, but I'm not sure that I can see Y&Z. And then there was the the the these schemers that produced hundreds and hundreds of thousands of production in X amount of days a year and you know it was as much of this **HOLDS UP A PEN/WAVES IT AROUND** than it was of the handpiece. You know what I mean? So it's like, and you were trying to do your best and you were trying to do the the kind of the honourable thing and keep the whole thing going. And then the scheme was running out of money and one of my colleagues in the Dental Council from the last term has you probably know who I'm talking about. Would would would have worked a whole lot on probity, and his book was excellent and there was a gaping need for for more probity. There was a gaping need for prosecution and this all through my career. And if they if the ‘powers that be’ had prosecuted, had made an example of of practitioners, the cake would have been, it would have been divided more equitably between people, the fees would have risen. But I I I, anecdotally, it's 10 to 20%, that's how much of this is is, is, is, is fraudulently claimed, etcetera, and this was a major problem and I think it probably still is a major problem and it doesn't it it, there has been various kind of efforts to to do this and there were some of which are are not grounded that well in, in, in, in law.*  *Because from the very earliest stages, even when we were trying to introduce, the we'll see the claiming form and things like that, you know, it was huge objections to any form of administration that was seen to be, you know, ‘checking on’, you know, the outflows and stuff like that. And I have to say there was the most minimal of actual… I was involved the the the the services board that control the funding, okay, it was in [TOWN], and it was absolutely minimal… the amount of checking that was going… it was very, very kind of minimum at that stage, right? It was, you know, basics…. checking teeth that were been treated that weren't treated previously on the database, stuff like that. But there was quite a lot of resistance…. for dentists it would be nor-, kind of, as we, normally, part of their…. makeup…to be resistant to administrative procedures (laughs) that were kind of, particularly looking at accountability.* |
| **6. Discussion**  The Discussion is coherent and appropriately situates the findings within Irish oral health reform and the broader international literature.  However, the opening section reiterates the programme theory in language closely aligned to the Results. Tightening this section would reduce repetition and allow clearer emphasis on the novel theoretical refinements beyond the earlier realist review.  The comparative discussion with England is insightful but would benefit from explicit caveats regarding cross-system comparability (differences in contract design, basket of care, oversight mechanisms).  The “Us and them” implications section is compelling and well articulated.  It could be further strengthened by translating each consolidated CMOC into more explicit, actionable system levers (e.g., contract design and remuneration reform, standardisation of prior approval governance, risk-based and peer-led QA approaches, reinstatement of consultative structures). Making explicit how these levers would disrupt negative mechanisms and activate countervailing mechanisms would enhance practical utility.  A small number of phrases read as evaluative. Framing these more explicitly as perceptions operating within a low-trust context would maintain analytic neutrality while preserving the strength of the findings. | *Many thanks for your suggestions which we agree will improve the flow of the manuscript.*  *We have removed the excess text in the opening discussion section, which we agree re-iterated much of the final programme theory.*  *Regarding the comparison of the NHS England GDS contract and Irish DTSS contract, we have added the following section at the end of that section:*  ***As highlighted above, the NHS GDS and DTSS contracts differ substantially in their design, baskets of available care, remuneration and oversight mechanisms. This limits the direct comparability of GDP experiences across the two systems and therefore interpretation of these cross-system comparisons should be undertaken with caution.***  *Regarding the ‘Us and them’ implications section, thank you for encouraging comments. To strengthen this further, we have included the following sentences which translate the CMOCs into actions based around health system levers (as per your suggestion):*  ***Sustained political and financial commitment to oral health, alongside greater integration of GDPs within public oral healthcare systems, may help counter the reinforcing mechanisms of marginalisation, isolation and mistrust which appear to underpin disengagement from state-funded care.***  *And*  ***More transparent and proportionate approaches to governance, communication and quality assurance may help shift perceptions of the health system among GDPs from punitive oversight towards supportive partnership, therefore countering defensiveness, mistrust and professional disengagement.***  **And**  ***The reinstatement of formal consultative and feedback structures, alongside more collaborative approaches to contract negotiation and system reform may help counter polarisation and foster reciprocity, shared ownership and greater trust between the profession and the health system.***  *Regarding the small number of phrases reading as evaluative, we have highlighted (with additional text added in green)in the limitations that:*  *“while interviews generated data which were, from a realist perspective, both contextually and mechanistically rich, they are representative of the opinions, attitudes and experiences of study participants and therefore can be highly subjective, as is common in qualitative methodological approaches (23). Unlike other realist studies, for example realist evaluations, which may triangulate qualitative findings with quantitative data or documentary analysis, this study was entirely reliant upon qualitative data and the perceived experiences, opinions and attitudes of interviewees,* ***many of which operated within a low-trust context*** *(37). Caution should therefore be exercised when interpreting these findings as the ultimate truth on this subject.”* |
| **7. Conclusion**  The conclusion succinctly summarises the key findings; however, it largely reiterates the identified CMOCs rather than distilling the central explanatory insight of the programme theory.  Consider tightening this section to foreground the core causal pathway — namely how political marginalisation, governance design, and communication practices interact to produce autonomy threat, mistrust, and disengagement within a mixed public–private market.  Framing mechanisms not solely as “sentiments” but as professional reasoning responses to structural conditions would further reinforce the realist explanatory contribution.  The conclusion could also more explicitly identify key modifiable system levers suggested by the findings (e.g., relational governance, supportive contract administration, consultative structures), strengthening its policy relevance. | *Many thanks for your suggestion on amending the conclusion section which, on reflection, we agree did reiterate much of the results. Taking on board your suggestions, we have amended the conclusion section to read as follows:*  ***This study identifies a clear causal pathway in which political marginalisation, restrictive governance, and adversarial communication practices shape GDPs’ professional reasoning about their role in public dental systems. Under these conditions, GDPs interpret system structures as constraining to their autonomy, undermining clinical judgement and triggering institutional mistrust. These interpretations, rather than diffuse sentiments, drive disengagement from state-funded oral healthcare systems, particularly within mixed public–private markets where private alternatives are perceived as professionally rewarding.***  ***Crucially, our findings highlight how modifiable system-level levers, namely governance processes, supportive and transparent contract administration, and meaningful consultative structures can potentially reshape how GDPs interpret system intent, mitigating perceived threats to autonomy and rebuilding trust.*** |

| **Reviewer #3** | |
| --- | --- |
| **Comment** | **Response** |
| To further strengthen the paper, greater transparency regarding the analytic process would be helpful with an audit table. | *Many thanks for taking the time to review our manuscript and for your suggestion on strengthening the paper through greater transparency on the analysis. While formal audit tables are not typically a feature of realist interview studies, we agree that further clarification of our analytic and refinement processes would strengthen the manuscript. We have therefore expanded the methods section to provide greater detail on how CMOCs were developed, refined, and consolidated throughout the study. Specifically, we now expand on our quality assurance (QA) processes which were adopted throughout the entire project. This namely entailed early and often sharing of data/results, regular meetings between the PI (lead author) and senior supervising co-authors to discuss emerging findings and the use of our external stakeholder advisory group. These QA processes were built into the research and were largely iterative and dynamic in nature (as opposed to those adopting quantitative metrics/kappa scoring etc). Additionally, we have added a section on reflexivity which we feel also adds to transparency.*  *To facilitate greater transparency and provide more detail on the analysis, we have amended the methods section by adding the following paragraphs in our methods section:*  ***The process of coding data, building, iteratively refining and crafting CMOCs took place through early and often sharing of emerging contexts, mechanisms and outcomes using a series of online shared documents (Microsoft Word and PowerPoint for data visualisation), and through regular meetings where CMOCs were discussed and refined. These processes, built in to the research served as a means of quality assurance were not quantitative, but rather iterative and dynamic in nature. This process resulted in 13 individual CMOCs presented in abstracted format. Thereafter, these were consolidated into 5 CMOCs specific to the Irish context and based around the broad themes or concepts under which these were felt to fall under by the research team.***  ***And***  ***Reflexivity***  ***A reflexive diary was maintained by the lead author after interviews. This facilitated reflective awareness of potential internal and external influences on data interpretation, including professional roles and the ethical implications of being a clinician-researcher. To support objectivity and minimise bias, the data were shared early and often with, and reviewed independently by senior supervising co-authors throughout the analysis and synthesis.*** |
| In addition, please consider including the full interview guide as a supplementary file to support transparency regarding the realist interviewing process. | *Please accept our apologies for not including this with the previous submission. The interview guide is now included in the supplemental material* |
| Finally, tables referenced in the manuscript and/or supplementary materials were not located; these should be provided or clearly indicated to ensure completeness of reporting. | *Please accept out apologies, the tables are now provided.* |
