# Supplementary material for: ‘Us and them’: A realist interview study exploring how and why health system factors influence dentists’ participation in state funded, contracted primary dental care for low-income populations in Ireland
Source: PLoS One. 2026 Jul 31;21(7):e0341786. doi: 10.1371/journal.pone.0341786 (PMC13426964; doi:10.1371/journal.pone.0341786)
Supplement: Peer review history

# Peer review: Decision Letter 0

**DOI:** 10.1371/journal.pone.0341786.r001

**Authors:** Helen Howard

9 Mar 2026

PONE-D-25-54593

‘Us and them’: a realist interview study exploring how and why health system factors influence dentists’ participation in state funded, contracted primary dental care for low-income populations in Ireland

PLOS One

Dear Dr. Leavy,

Thank you for submitting your manuscript to PLOS ONE. After careful consideration, we feel that it has merit but does not fully meet PLOS ONE’s publication criteria as it currently stands. Therefore, we invite you to submit a revised version of the manuscript that addresses the points raised during the review process.

=============================

The manuscript has been evaluated by three reviewers, and their comments are available below.

The reviewers have raised a number of concerns that need attention. In particular, they request additional information on methodological aspects of the study, further details on reflexivity, and improvements to the Discussion section.

Could you please revise the manuscript to carefully address the concerns raised?

=============================

Please submit your revised manuscript by Apr 22 2026 11:59PM. If you will need more time than this to complete your revisions, please reply to this message or contact the journal office at plosone@plos.org. When you're ready to submit your revision, log on to https://www.editorialmanager.com/pone/ and select the 'Submissions Needing Revision' folder to locate your manuscript file.

Please include the following items when submitting your revised manuscript:

- A letter that responds to each point raised by the academic editor and reviewer(s). You should upload this letter as a separate file labeled 'Response to Reviewers'.
- A marked-up copy of your manuscript that highlights changes made to the original version. You should upload this as a separate file labeled 'Revised Manuscript with Track Changes'.
- An unmarked version of your revised paper without tracked changes. You should upload this as a separate file labeled 'Manuscript'.

If you would like to make changes to your financial disclosure, please include your updated statement in your cover letter. Guidelines for resubmitting your figure files are available below the reviewer comments at the end of this letter.

If applicable, we recommend that you deposit your laboratory protocols in protocols.io to enhance the reproducibility of your results. Protocols.io assigns your protocol its own identifier (DOI) so that it can be cited independently in the future. For instructions see: https://journals.plos.org/plosone/s/submission-guidelines#loc-laboratory-protocols. Additionally, PLOS ONE offers an option for publishing peer-reviewed Lab Protocol articles, which describe protocols hosted on protocols.io. Read more information on sharing protocols at https://plos.org/protocols?utm_medium=editorial-email&utm_source=authorletters&utm_campaign=protocols.

We look forward to receiving your revised manuscript.

Kind regards,

Helen Howard

Staff Editor

PLOS One

Journal Requirements:

When submitting your revision, we need you to address these additional requirements.

1. Please ensure that your manuscript meets PLOS ONE's style requirements, including those for file naming. The PLOS ONE style templates can be found at

https://journals.plos.org/plosone/s/file?id=wjVg/PLOSOne_formatting_sample_main_body.pdf and https://journals.plos.org/plosone/s/file?id=ba62/PLOSOne_formatting_sample_title_authors_affiliations.pdf

2. We note that you have indicated that there are restrictions to data sharing for this study. For studies involving human research participant data or other sensitive data, we encourage authors to share de-identified or anonymized data. However, when data cannot be publicly shared for ethical reasons, we allow authors to make their data sets available upon request. For information on unacceptable data access restrictions, please see http://journals.plos.org/plosone/s/data-availability#loc-unacceptable-data-access-restrictions.

Before we proceed with your manuscript, please address the following prompts:

a) If there are ethical or legal restrictions on sharing a de-identified data set, please explain them in detail (e.g., data contain potentially identifying or sensitive patient information, data are owned by a third-party organization, etc.) and who has imposed them (e.g., a Research Ethics Committee or Institutional Review Board, etc.). Please also provide contact information for a data access committee, ethics committee, or other institutional body to which data requests may be sent.

b) If there are no restrictions, please upload the minimal anonymized data set necessary to replicate your study findings to a stable, public repository and provide us with the relevant URLs, DOIs, or accession numbers. Please see http://www.bmj.com/content/340/bmj.c181.long for guidelines on how to de-identify and prepare clinical data for publication. For a list of recommended repositories, please see https://journals.plos.org/plosone/s/recommended-repositories. You also have the option of uploading the data as Supporting Information files, but we would recommend depositing data directly to a data repository if possible.

Please update your Data Availability statement in the submission form accordingly.

3. When completing the data availability statement of the submission form, you indicated that you will make your data available on acceptance. We strongly recommend all authors decide on a data sharing plan before acceptance, as the process can be lengthy and hold up publication timelines. Please note that, though access restrictions are acceptable now, your entire data will need to be made freely accessible if your manuscript is accepted for publication. This policy applies to all data except where public deposition would breach compliance with the protocol approved by your research ethics board. If you are unable to adhere to our open data policy, please kindly revise your statement to explain your reasoning and we will seek the editor's input on an exemption. Please be assured that, once you have provided your new statement, the assessment of your exemption will not hold up the peer review process.

4. Thank you for stating the following financial disclosure:

Research funded by the Health Research Board SPHeRE 2018-1

Please state what role the funders took in the study. If the funders had no role, please state: "The funders had no role in study design, data collection and analysis, decision to publish, or preparation of the manuscript."

If this statement is not correct you must amend it as needed.

Please include this amended Role of Funder statement in your cover letter; we will change the online submission form on your behalf.

5. Thank you for stating the following in the Acknowledgments Section of your manuscript:

The authors wish to thank the following members of the Project Advisory Group (PAG): Professor Paul Batchelor, Dr Sarah Barry, Dr Will Rymer, Dr. Katherine Carr, Mr Michael Dolan, Miss Sophie Mulcahy Symmons. This Research is funded by the Irish Health Research Board (HRB) under the SPHeRE Programme 2018-1

We note that you have provided funding information that is not currently declared in your Funding Statement. However, funding information should not appear in the Acknowledgments section or other areas of your manuscript. We will only publish funding information present in the Funding Statement section of the online submission form.

Please remove any funding-related text from the manuscript and let us know how you would like to update your Funding Statement. Currently, your Funding Statement reads as follows:

Research funded by the Health Research Board SPHeRE 2018-1

Please include your amended statements within your cover letter; we will change the online submission form on your behalf.

6. Please include a copy of Table 2 which you refer to in your text on page 14.

7. We note you have included a table to which you do not refer in the text of your manuscript. Please ensure that you refer to Table 3 in your text; if accepted, production will need this reference to link the reader to the Table.

8. Please include captions for your Supporting Information files at the end of your manuscript, and update any in-text citations to match accordingly. Please see our Supporting Information guidelines for more information: http://journals.plos.org/plosone/s/supporting-information.

9. If the reviewer comments include a recommendation to cite specific previously published works, please review and evaluate these publications to determine whether they are relevant and should be cited. There is no requirement to cite these works unless the editor has indicated otherwise.

[Note: HTML markup is below. Please do not edit.]

Reviewers' comments:

Reviewer's Responses to Questions

**Comments to the Author**

1. Is the manuscript technically sound, and do the data support the conclusions?

The manuscript must describe a technically sound piece of scientific research with data that supports the conclusions. Experiments must have been conducted rigorously, with appropriate controls, replication, and sample sizes. The conclusions must be drawn appropriately based on the data presented.

Reviewer #1: Yes

Reviewer #2: Yes

Reviewer #3: Yes

**********

2. Has the statistical analysis been performed appropriately and rigorously?

Reviewer #1: N/A

Reviewer #2: Yes

Reviewer #3: N/A

**********

3. Have the authors made all data underlying the findings in their manuscript fully available?

The PLOS Data policy requires authors to make all data underlying the findings described in their manuscript fully available without restriction, with rare exception (please refer to the Data Availability Statement in the manuscript PDF file). The data should be provided as part of the manuscript or its supporting information, or deposited to a public repository. For example, in addition to summary statistics, the data points behind means, medians and variance measures should be available. If there are restrictions on publicly sharing data—e.g. participant privacy or use of data from a third party—those must be specified.

Reviewer #1: Yes

Reviewer #2: Yes

Reviewer #3: No

**********

4. Is the manuscript presented in an intelligible fashion and written in standard English?

PLOS ONE does not copyedit accepted manuscripts, so the language in submitted articles must be clear, correct, and unambiguous. Any typographical or grammatical errors should be corrected at revision, so please note any specific errors here.

Reviewer #1: Yes

Reviewer #2: Yes

Reviewer #3: Yes

**********

5. Review Comments to the Author

Please use the space provided to explain your answers to the questions above. You may also include additional comments for the author, including concerns about dual publication, research ethics, or publication ethics. (Please upload your review as an attachment if it exceeds 20,000 characters)

Reviewer #1: This is a brilliant piece of scholarship filling a gap in the global literature. This study examines the health system factors that are likely structurally causative to dentist non-participation in public programs, and therefore provides a roadmap of political/structural/policy approaches that could remediate the situation and improve access to dental care both in Ireland and around the globe. It is well written, empirically sound, and based in the literature. No substantive critiques - excellent article.

Reviewer #2: First, I would like to congratulate the authors on a well-developed and methodologically sound study addressing an important and policy-relevant issue. The use of realist interviews to examine health system influences on GDP engagement in state-funded care is both appropriate and timely. The manuscript demonstrates strong conceptual grounding, adherence to realist principles, and clear policy relevance within the Irish reform context.

The study clearly addresses a significant knowledge gap and builds meaningfully upon the authors’ prior realist review.

That said, I offer the following suggestions to strengthen clarity, methodological transparency, and theoretical contribution.

1. Introduction and Rationale

There is some redundancy between the “Study rationale” section in the Introduction and the subsequent “Rationale” subsection within the Methods. The justification for a realist approach appears twice — first conceptually in the Introduction and then methodologically in the Methods.

It may improve narrative flow to more clearly differentiate these sections:

The Introduction should justify why the study is needed (policy and empirical gap).

The Methods should justify why a realist approach is appropriate and how it was operationalised.

Streamlining this would reduce repetition and strengthen structural coherence.

2. Sampling and Theoretical Sufficiency

The inclusion of diverse stakeholders is a notable strength. However, clarification is needed regarding:

Why only six frontline GDPs were included.

Whether this number was determined by theoretical sufficiency.

How “theoretical sufficiency” was operationalised in the context of realist interviews.

It would be helpful if the authors could elaborate on:

How they determined that sufficient variation had been achieved,

Whether new mechanisms ceased to emerge,

And how sufficiency was judged within the iterative realist interview process.

3. Reflexivity

Given the theory-driven nature of realist interviews, reflexivity is particularly important. The manuscript would benefit from clarification regarding:

How the interviewer’s prior knowledge and involvement in the earlier realist review may have influenced the interview process.

Whether reflexive journaling or other strategies were used to monitor interpretive influence.

4. CMOC Development and Refinement

Further methodological transparency would strengthen the realist contribution. Specifically:

Were CMOCs refined iteratively across interviews?

Were conflicting or competing CMOCs retained and explored?

How were the 13 individual CMOCs consolidated into five higher-level CMOCs?

Providing a brief explanation of this consolidation process would enhance analytic clarity.

5. Results – Differentiation of Context, Mechanism, and Outcome

The Results are rich and policy-relevant, and the five consolidated CMOCs form a coherent explanatory account.

To further strengthen the realist contribution, the manuscript would benefit from clearer and more consistent differentiation between:

Contexts, Mechanisms (reasoning/resources), and Outcomes.

Several constructs (e.g., trust, demotivation, fragmentation) appear to function in multiple roles across CMOCs. Clarifying how these were categorised would improve conceptual precision.

Additionally, some potentially contentious claims (e.g., references to cultural opportunism or perceptions of accusatory tone) would be strengthened by clarifying:

Whether these views were widely shared or contested across stakeholder groups, and

How disconfirming or divergent cases informed programme theory refinement.

6. Discussion

The Discussion is coherent and appropriately situates the findings within Irish oral health reform and the broader international literature.

However, the opening section reiterates the programme theory in language closely aligned to the Results. Tightening this section would reduce repetition and allow clearer emphasis on the novel theoretical refinements beyond the earlier realist review.

The comparative discussion with England is insightful but would benefit from explicit caveats regarding cross-system comparability (differences in contract design, basket of care, oversight mechanisms).

The “Us and them” implications section is compelling and well articulated. It could be further strengthened by translating each consolidated CMOC into more explicit, actionable system levers (e.g., contract design and remuneration reform, standardisation of prior approval governance, risk-based and peer-led QA approaches, reinstatement of consultative structures). Making explicit how these levers would disrupt negative mechanisms and activate countervailing mechanisms would enhance practical utility.

A small number of phrases read as evaluative. Framing these more explicitly as perceptions operating within a low-trust context would maintain analytic neutrality while preserving the strength of the findings.

7. Conclusion

The conclusion succinctly summarises the key findings; however, it largely reiterates the identified CMOCs rather than distilling the central explanatory insight of the programme theory.

Consider tightening this section to foreground the core causal pathway — namely how political marginalisation, governance design, and communication practices interact to produce autonomy threat, mistrust, and disengagement within a mixed public–private market.

Framing mechanisms not solely as “sentiments” but as professional reasoning responses to structural conditions would further reinforce the realist explanatory contribution. The conclusion could also more explicitly identify key modifiable system levers suggested by the findings (e.g., relational governance, supportive contract administration, consultative structures), strengthening its policy relevance.

Reviewer #3: This is a well-written and thoughtfully conducted study addressing an important topic, and the manuscript demonstrates considerable effort in applying a realist approach utilising the teaching-learning cycle, WHO health systems building blocks and program theory frameworks. To further strengthen the paper, greater transparency regarding the analytic process would be helpful with an audit table. In addition, please consider including the full interview guide as a supplementary file to support transparency regarding the realist interviewing process. Finally, tables referenced in the manuscript and/or supplementary materials were not located; these should be provided or clearly indicated to ensure completeness of reporting.

**********

6. PLOS authors have the option to publish the peer review history of their article (what does this mean?). If published, this will include your full peer review and any attached files.

If you choose “no”, your identity will remain anonymous but your review may still be made public.

**Do you want your identity to be public for this peer review?**  For information about this choice, including consent withdrawal, please see our Privacy Policy.

Reviewer #1: No

Reviewer #2: **Yes:** Vineetha Karuveettil

Reviewer #3: No

**********

[NOTE: If reviewer comments were submitted as an attachment file, they will be attached to this email and accessible via the submission site. Please log into your account, locate the manuscript record, and check for the action link "View Attachments". If this link does not appear, there are no attachment files.]

To ensure your figures meet our technical requirements, please review our figure guidelines: https://journals.plos.org/plosone/s/figures

You may also use PLOS’s free figure tool, NAAS, to help you prepare publication quality figures: https://journals.plos.org/plosone/s/figures#loc-tools-for-figure-preparation.

NAAS will assess whether your figures meet our technical requirements by comparing each figure against our figure specifications.

# Author comment: Author response to Decision Letter 1

**DOI:** 10.1371/journal.pone.0341786.r002

12 May 2026

Dear Reviewers,

Many thanks for taking the time to review our manuscript and for your thoughtful, encouraging and constructive commentary. We have taken the time to consider each of the comments careful and we have endeavoured to amend the manuscript accordingly.

Please see the attached separate word document which was uploaded and contains a detailed table responding to each of the reviewers comments.

Yours sincerely,

Research Team

Attachment Submitted filename: Response to Reviewers.docx

# Peer review: Decision Letter 1

**DOI:** 10.1371/journal.pone.0341786.r003

**Authors:** Maria Giulia Nosotti

18 Jun 2026

‘Us and them’: a realist interview study exploring how and why health system factors influence dentists’ participation in state funded, contracted primary dental care for low-income populations in Ireland

PONE-D-25-54593R1

Dear Dr. Paul Leavy,

We’re pleased to inform you that your manuscript has been judged scientifically suitable for publication and will be formally accepted for publication once it meets all outstanding technical requirements.

Within one week, you’ll receive an e-mail detailing the required amendments. When these have been addressed, you’ll receive a formal acceptance letter and your manuscript will be scheduled for publication.

An invoice will be generated when your article is formally accepted. Please note, if your institution has a publishing partnership with PLOS and your article meets the relevant criteria, all or part of your publication costs will be covered. Please make sure your user information is up-to-date by logging into Editorial Manager at Editorial Manager® and clicking the ‘Update My Information' link at the top of the page. For questions related to billing, please contact billing support.

If your institution or institutions have a press office, please notify them about your upcoming paper to help maximize its impact. If they’ll be preparing press materials, please inform our press team as soon as possible -- no later than 48 hours after receiving the formal acceptance. Your manuscript will remain under strict press embargo until 2 pm Eastern Time on the date of publication. For more information, please contact onepress@plos.org.

Kind regards,

Maria Giulia Nosotti, Master's Degree

Academic Editor

PLOS One

Additional Editor Comments (optional):

Reviewers' comments:

Reviewer's Responses to Questions

**Comments to the Author**

1. If the authors have adequately addressed your comments raised in a previous round of review and you feel that this manuscript is now acceptable for publication, you may indicate that here to bypass the “Comments to the Author” section, enter your conflict of interest statement in the “Confidential to Editor” section, and submit your "Accept" recommendation.

Reviewer #2: All comments have been addressed

Reviewer #3: All comments have been addressed

**********

2. Is the manuscript technically sound, and do the data support the conclusions?

The manuscript must describe a technically sound piece of scientific research with data that supports the conclusions. Experiments must have been conducted rigorously, with appropriate controls, replication, and sample sizes. The conclusions must be drawn appropriately based on the data presented.

Reviewer #2: Yes

Reviewer #3: Yes

**********

3. Has the statistical analysis been performed appropriately and rigorously?

Reviewer #2: Yes

Reviewer #3: N/A

**********

4. Have the authors made all data underlying the findings in their manuscript fully available?

The PLOS Data policy requires authors to make all data underlying the findings described in their manuscript fully available without restriction, with rare exception (please refer to the Data Availability Statement in the manuscript PDF file). The data should be provided as part of the manuscript or its supporting information, or deposited to a public repository. For example, in addition to summary statistics, the data points behind means, medians and variance measures should be available. If there are restrictions on publicly sharing data—e.g. participant privacy or use of data from a third party—those must be specified.

Reviewer #2: Yes

Reviewer #3: Yes

**********

5. Is the manuscript presented in an intelligible fashion and written in standard English?

PLOS ONE does not copyedit accepted manuscripts, so the language in submitted articles must be clear, correct, and unambiguous. Any typographical or grammatical errors should be corrected at revision, so please note any specific errors here.

Reviewer #2: Yes

Reviewer #3: Yes

**********

6. Review Comments to the Author

Please use the space provided to explain your answers to the questions above. You may also include additional comments for the author, including concerns about dual publication, research ethics, or publication ethics. (Please upload your review as an attachment if it exceeds 20,000 characters)

Reviewer #2: The authors have adequately addressed all the comments and suggestions raised during the review process. The revised manuscript reflects the necessary modifications and improvements in clarity, methodology, and presentation of results. The responses provided by the authors are satisfactory and demonstrate careful consideration of the reviewers’ concerns. Overall, the updated manuscript is well-structured, scientifically sound, and suitable for publication in its present form.

Reviewer #3: I feel the authors have addressed all questions adequately. However I did not see the interview guide or the tables that have been referenced in the manuscript.

**********

7. PLOS authors have the option to publish the peer review history of their article (what does this mean?). If published, this will include your full peer review and any attached files.

If you choose “no”, your identity will remain anonymous but your review may still be made public.

**Do you want your identity to be public for this peer review?**  For information about this choice, including consent withdrawal, please see our Privacy Policy.

Reviewer #2: **Yes:** Vineetha Karuveettil

Reviewer #3: No

**********

# Editor report: Acceptance letter

**DOI:** 10.1371/journal.pone.0341786.r004

**Authors:** Maria Giulia Nosotti

PONE-D-25-54593R1

PLOS One

Dear Dr. Leavy,

I'm pleased to inform you that your manuscript has been deemed suitable for publication in PLOS One. Congratulations! Your manuscript is now being handed over to our production team.

At this stage, our production department will prepare your paper for publication. This includes ensuring the following:

* All references, tables, and figures are properly cited

* All relevant supporting information is included in the manuscript submission,

* There are no issues that prevent the paper from being properly typeset

You will receive further instructions from the production team, including instructions on how to review your proof when it is ready. Please keep in mind that we are working through a large volume of accepted articles, so please give us a few days to review your paper and let you know the next and final steps.

Lastly, if your institution or institutions have a press office, please let them know about your upcoming paper now to help maximize its impact. If they'll be preparing press materials, please inform our press team within the next 48 hours. Your manuscript will remain under strict press embargo until 2 pm Eastern Time on the date of publication. For more information, please contact onepress@plos.org.

You will receive an invoice from PLOS for your publication fee after your manuscript has reached the completed accept phase. If you receive an email requesting payment before acceptance or for any other service, this may be a phishing scheme. Learn how to identify phishing emails and protect your accounts at https://explore.plos.org/phishing.

If we can help with anything else, please email us at customercare@plos.org.

Thank you for submitting your work to PLOS ONE and supporting open access.

Kind regards,

PLOS ONE Editorial Office Staff

on behalf of

Dr. Maria Giulia Nosotti

Academic Editor

PLOS One
